# Supplementary material for: Fluorescent Azasteroids through Ultrasound Assisted Cycloaddition Reactions
Source: Molecules. 2021 Aug 23;26(16):5098. doi: 10.3390/molecules26165098 (PMC8400541; doi:10.3390/molecules26165098)
Supplement: Supplementary file 1 [file molecules-26-05098-s001.zip › molecules-1335127 supplementary.pdf]

Supporting information for:

Fluorescent azasteroids through ultrasound assisted cycloaddition reactions

Costel Moldoveanu [1, *], Ionel Mangalagiu [1,2] and Gheorghita Zbancioc [1,*]

[1] Chemistry Department, Alexandru Ioan Cuza University of Iasi, 11 Carol 1st Bvd, Iasi -700506, Romania; ionelm@uaic.ro (I.M.)
[2] Integrated Centre of Environmental Science Studies in the North Eastern Region (CERNESIM), Alexandru Ioan Cuza University of Iasi, 11 Carol I, Iasi, 700506, Romania; ionelm@uaic.ro (I.M.)
* Correspondence: costel.moldoveanu@uaic.ro (C.M.); gheorghita.zbancioc@uaic.ro (G.Z.) Tel.: +40-232-201278

Contents

1. NMR spectra of the obtained compounds..2
2. IR spectra of the obtained compounds. 12
3. ^{1}H-NMR studies on compound 7a at room temperature. 16
4. UV spectra of the obtained compounds..17
5. Emission spectra of the obtained compounds..24
6. Absorption vs emission spectra of the obtained compounds..31

1. NMR spectra of the obtained compounds.

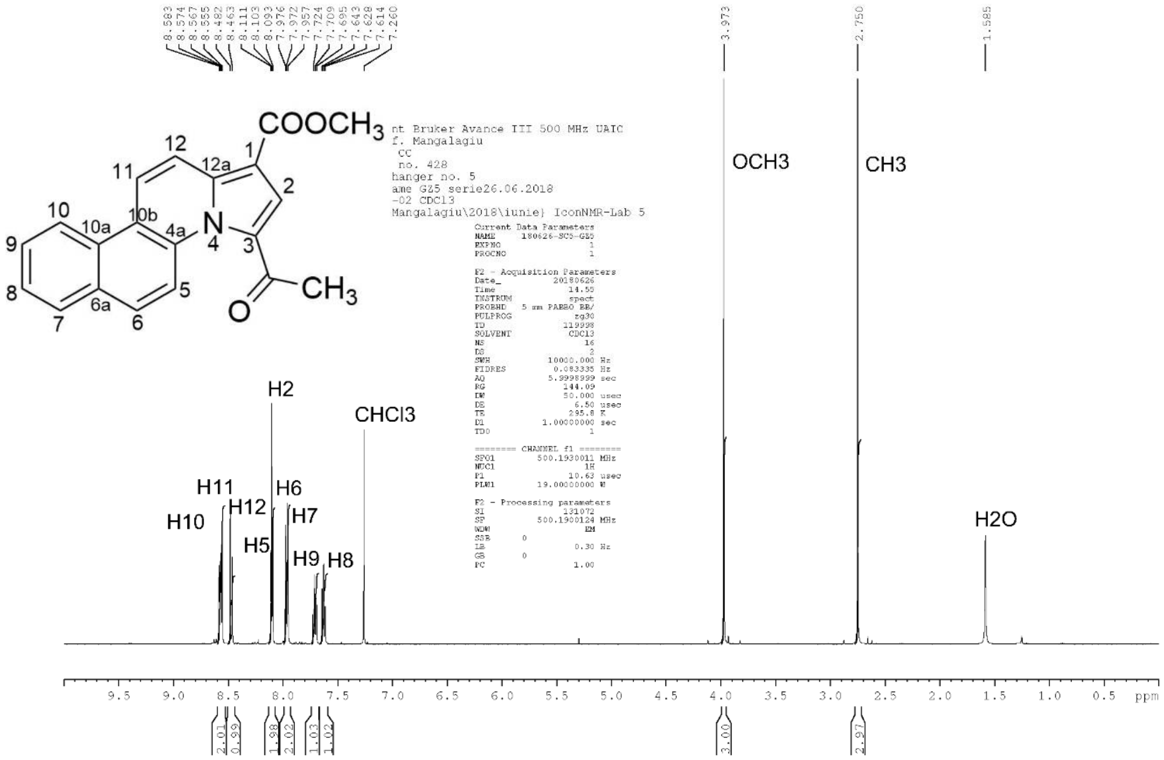

Figure S1a. ^{1}H NMR spectrum of the compound **5a.**

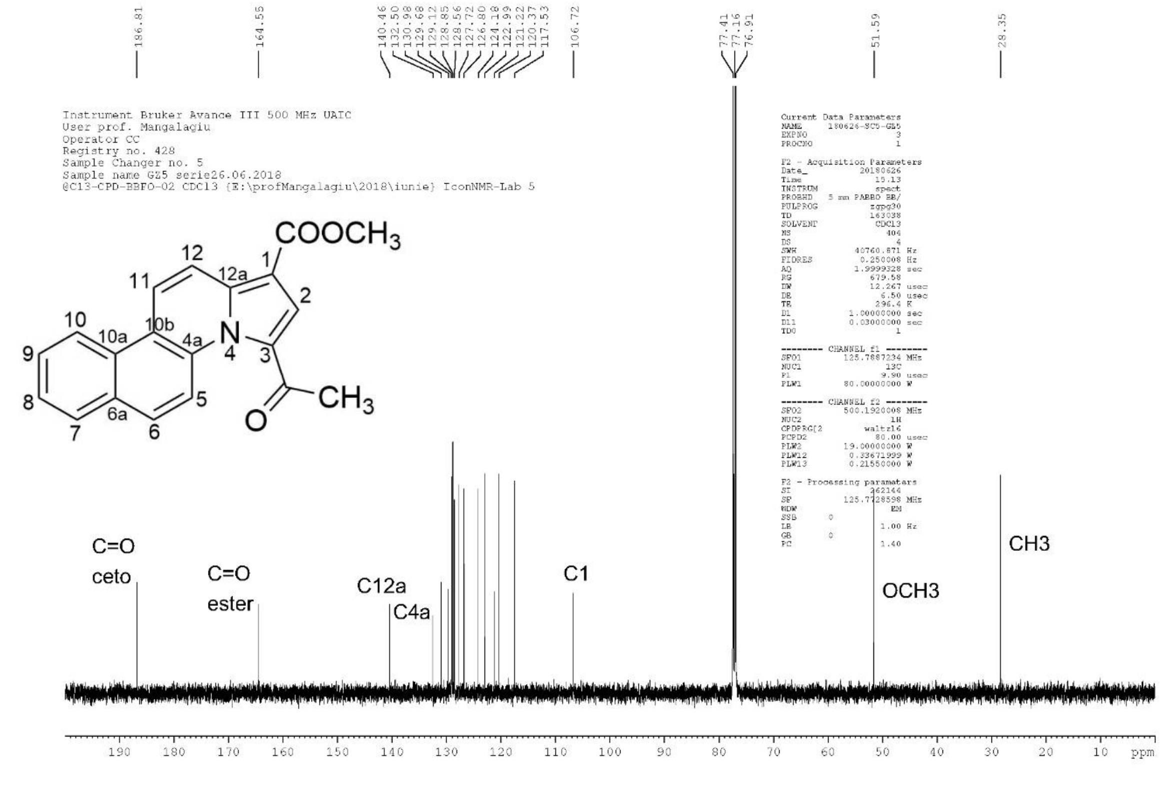

Figure S1b. ^{13}C NMR spectrum of the compound **5a.**

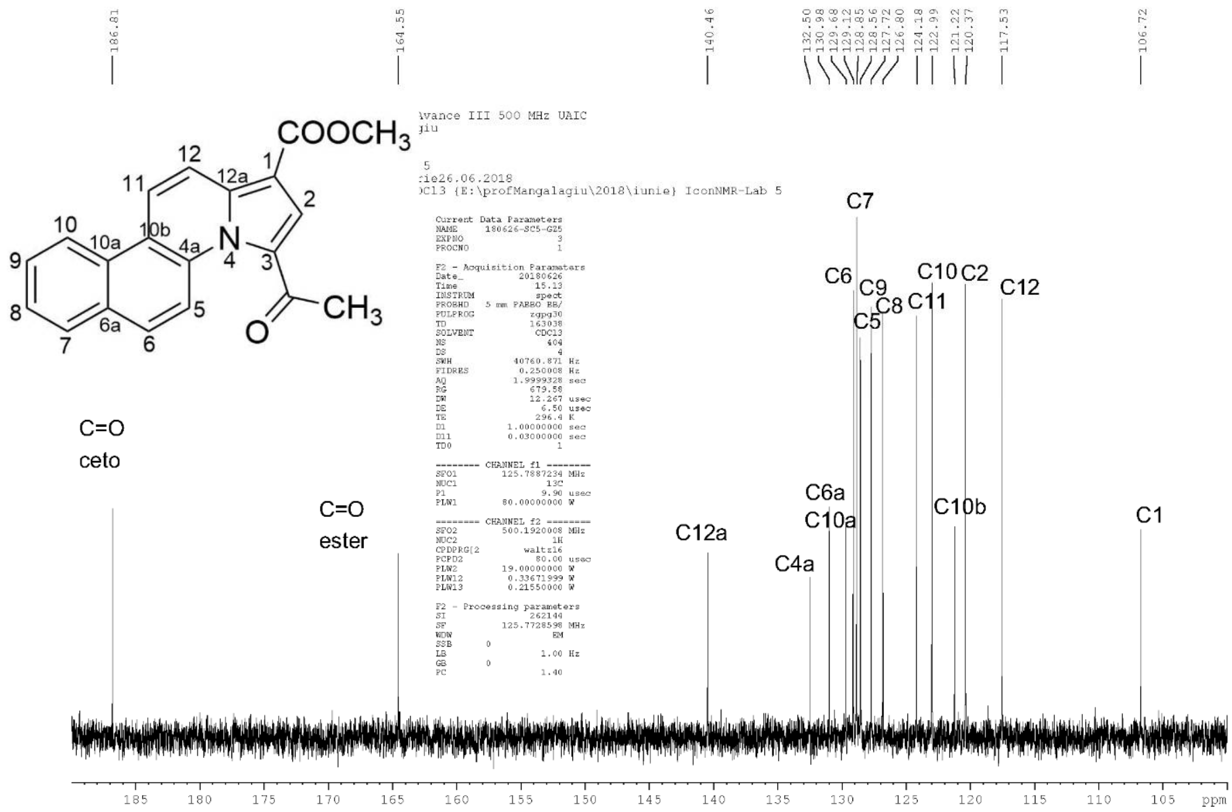

Figure S1c. Detail in the aromatic area of the ^{13}C NMR spectrum of the compound **5a.**

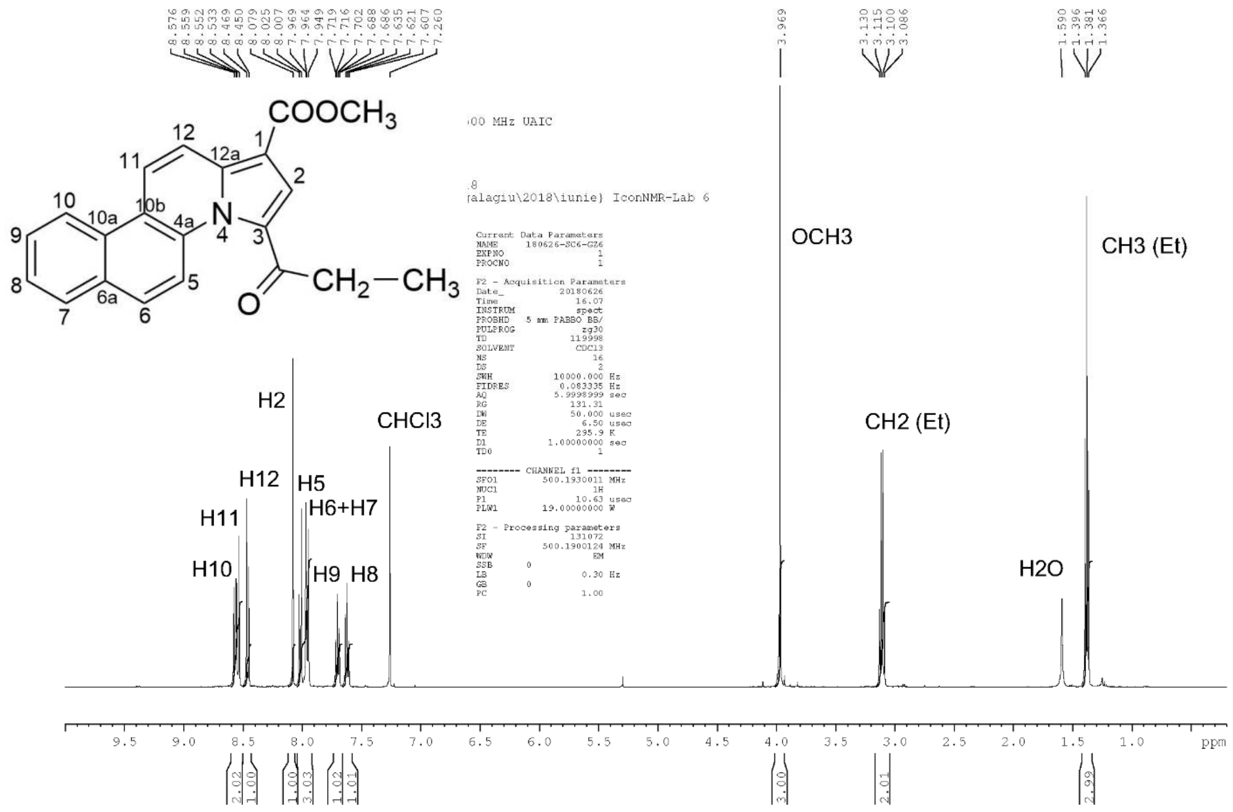

Figure S2a. ^{1}H NMR spectrum of the compound **5b.**

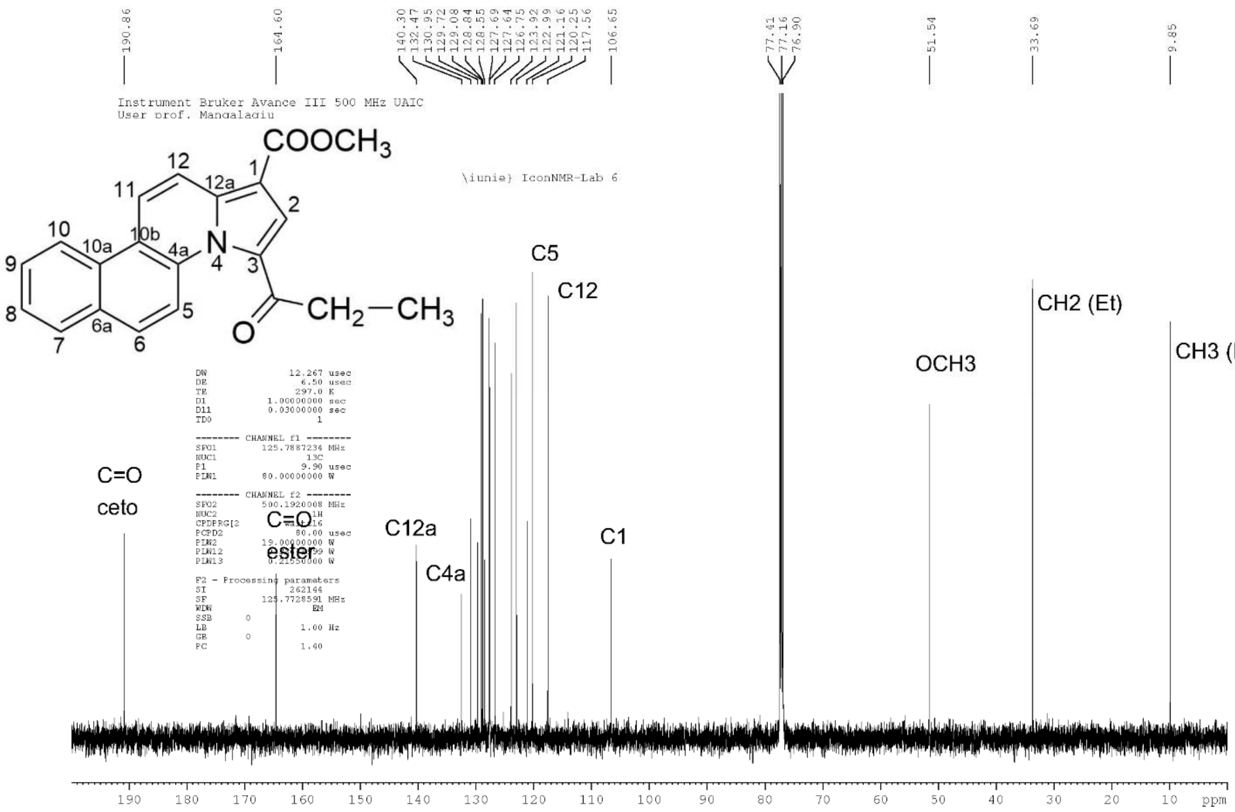

Figure S2b. ¹³C NMR spectrum of the compound **5b.**

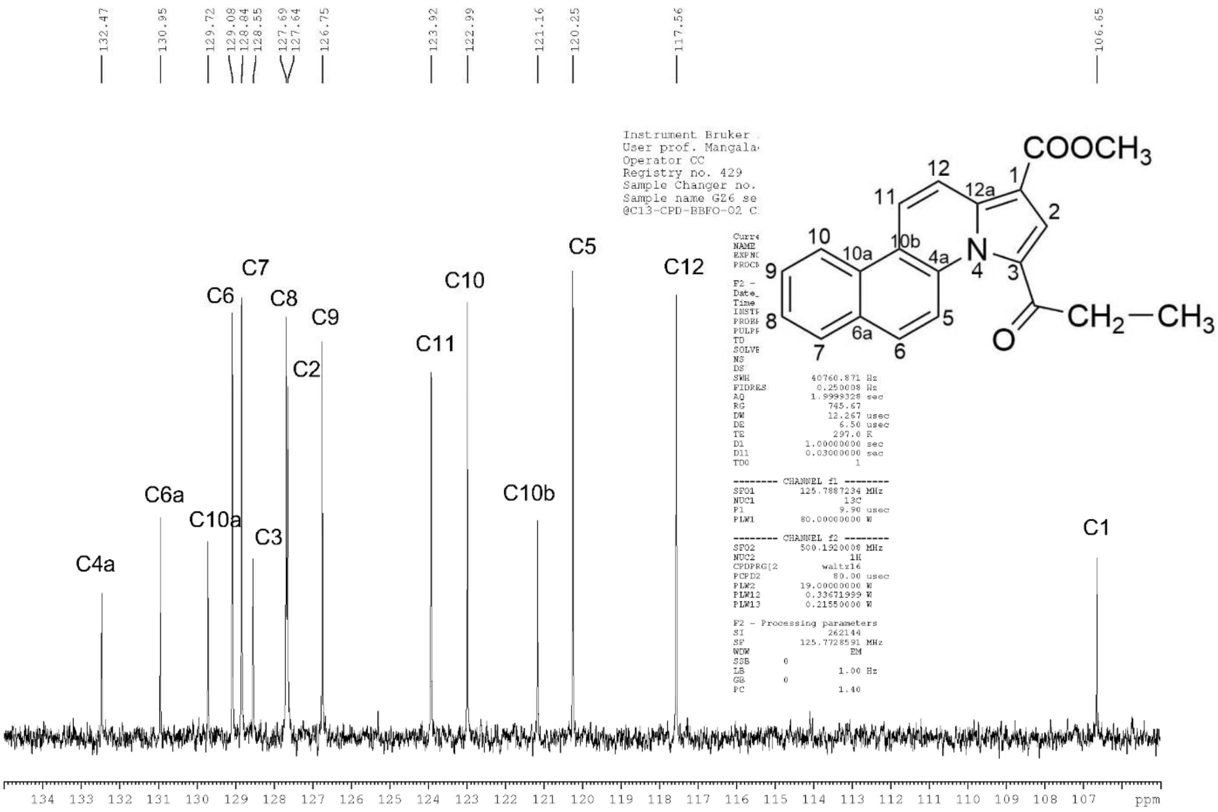

Figure S2c. Detail in the aromatic area of the ¹³C NMR spectrum of the compound **5b.**

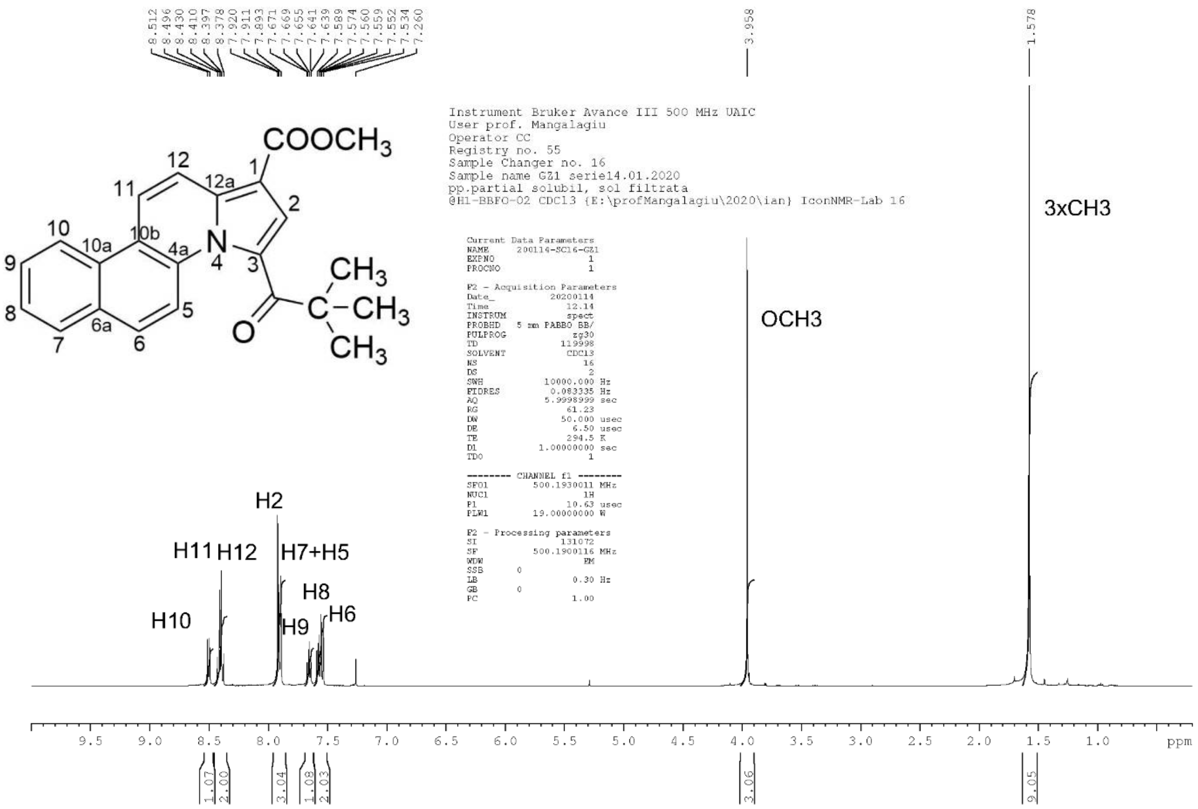

Figure S3a. ^{1}H NMR spectrum of the compound **5c.**

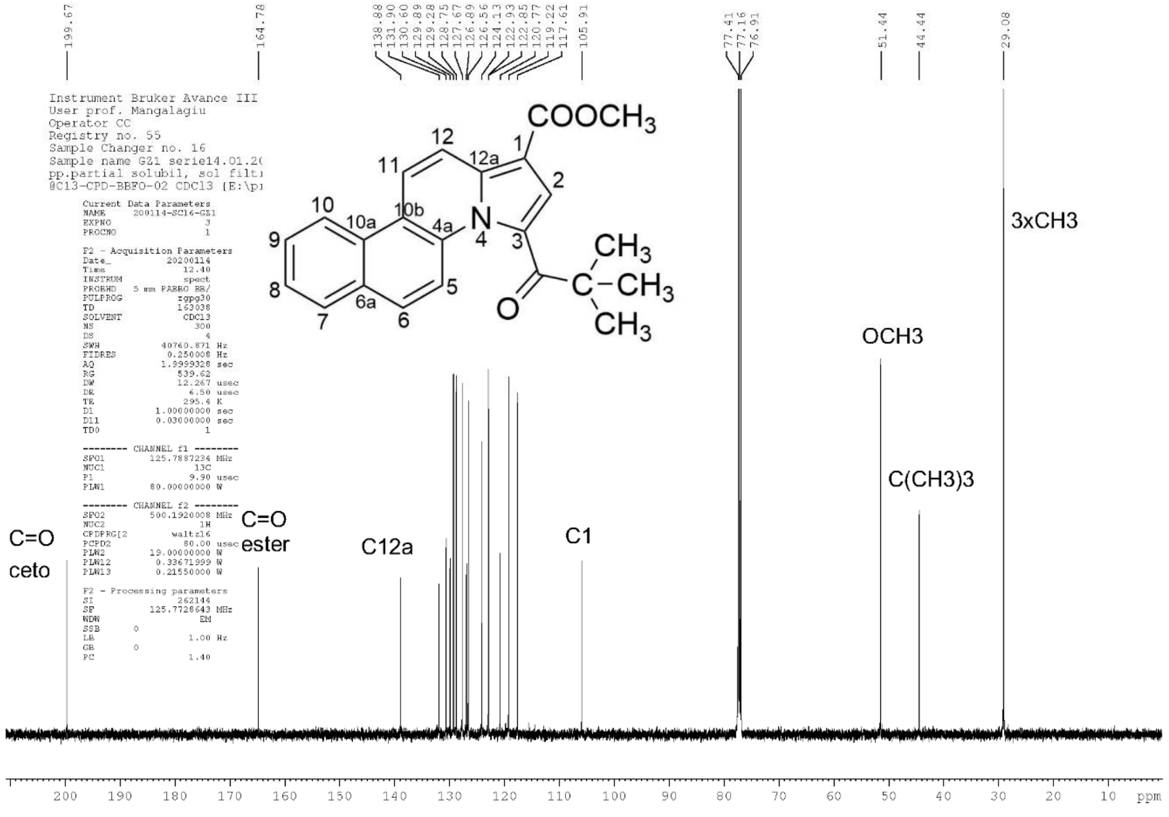

Figure S3b. ^{13}C NMR spectrum of the compound **5c.**

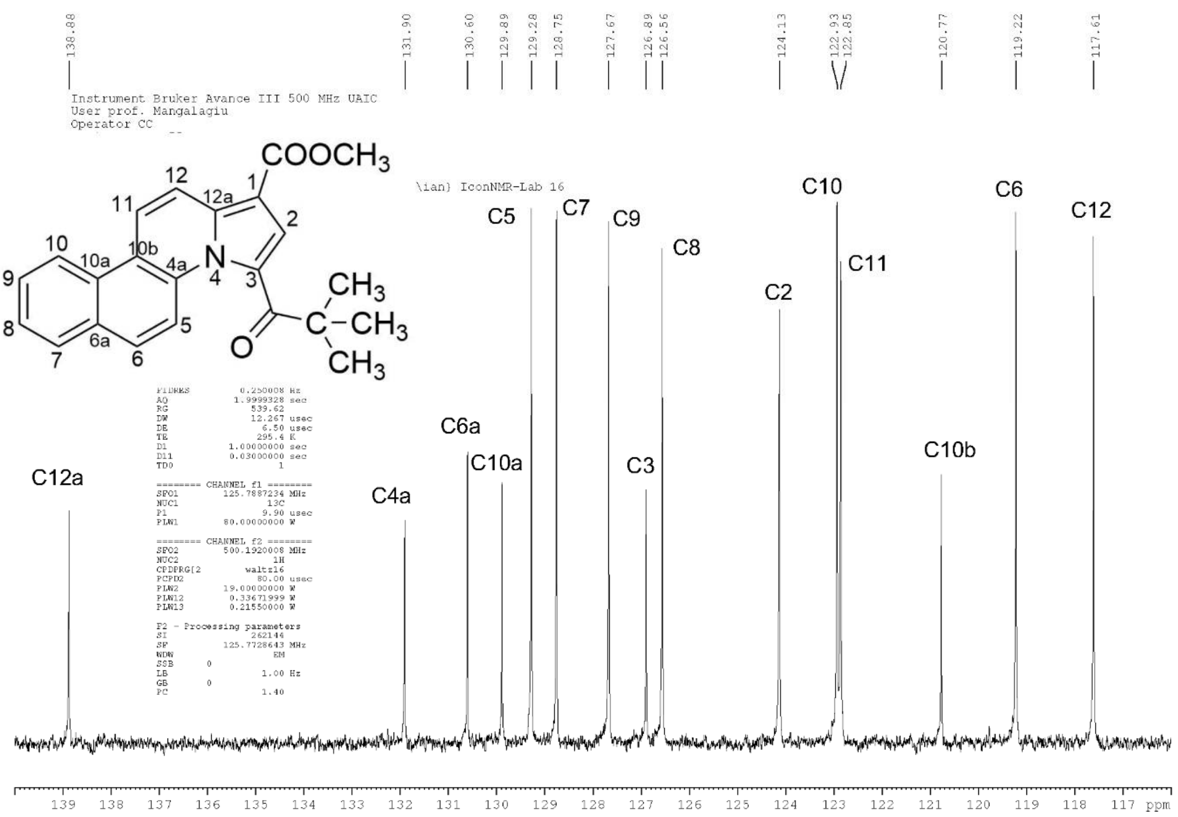

Figure S3c. Detail in the aromatic area of the ^{13}C NMR spectrum of the compound **5c.**

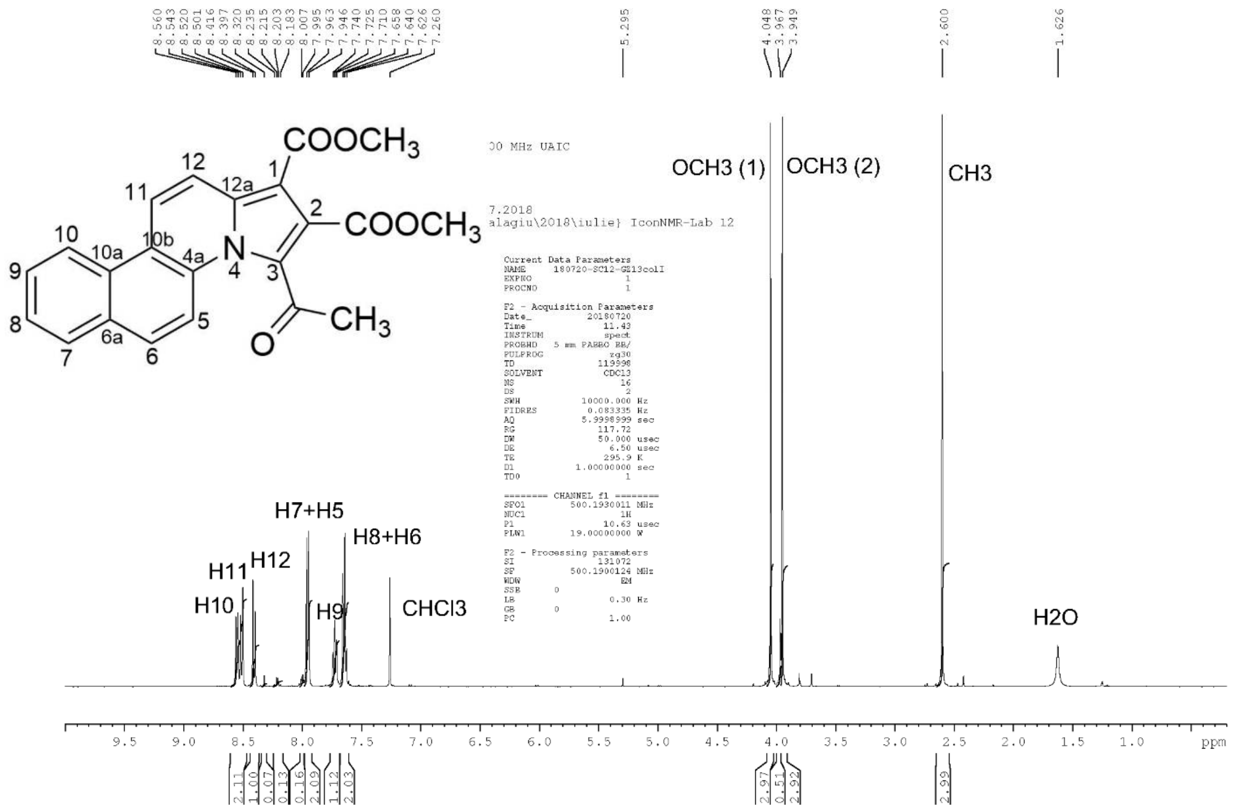

Figure S4a. ^{1}H NMR spectrum of the compound **7a.**

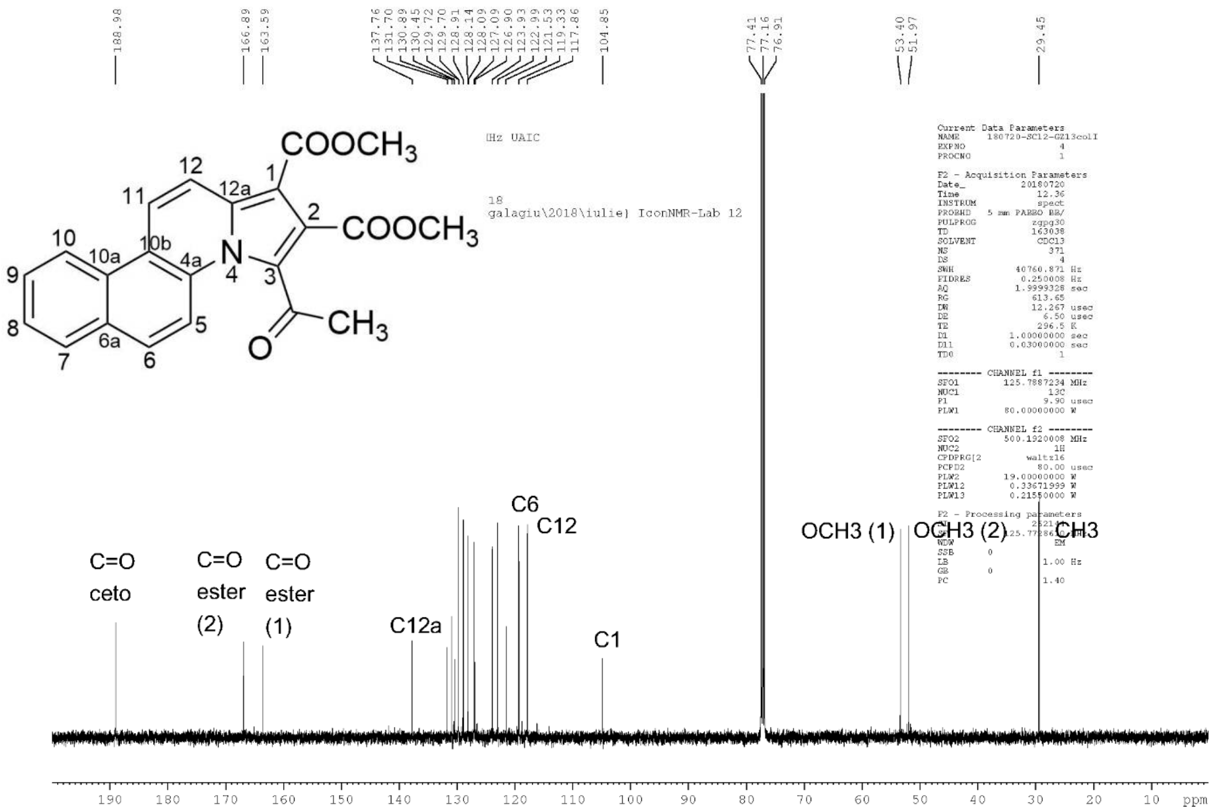

Figure S4b. ^{13}C NMR spectrum of the compound **7a.**

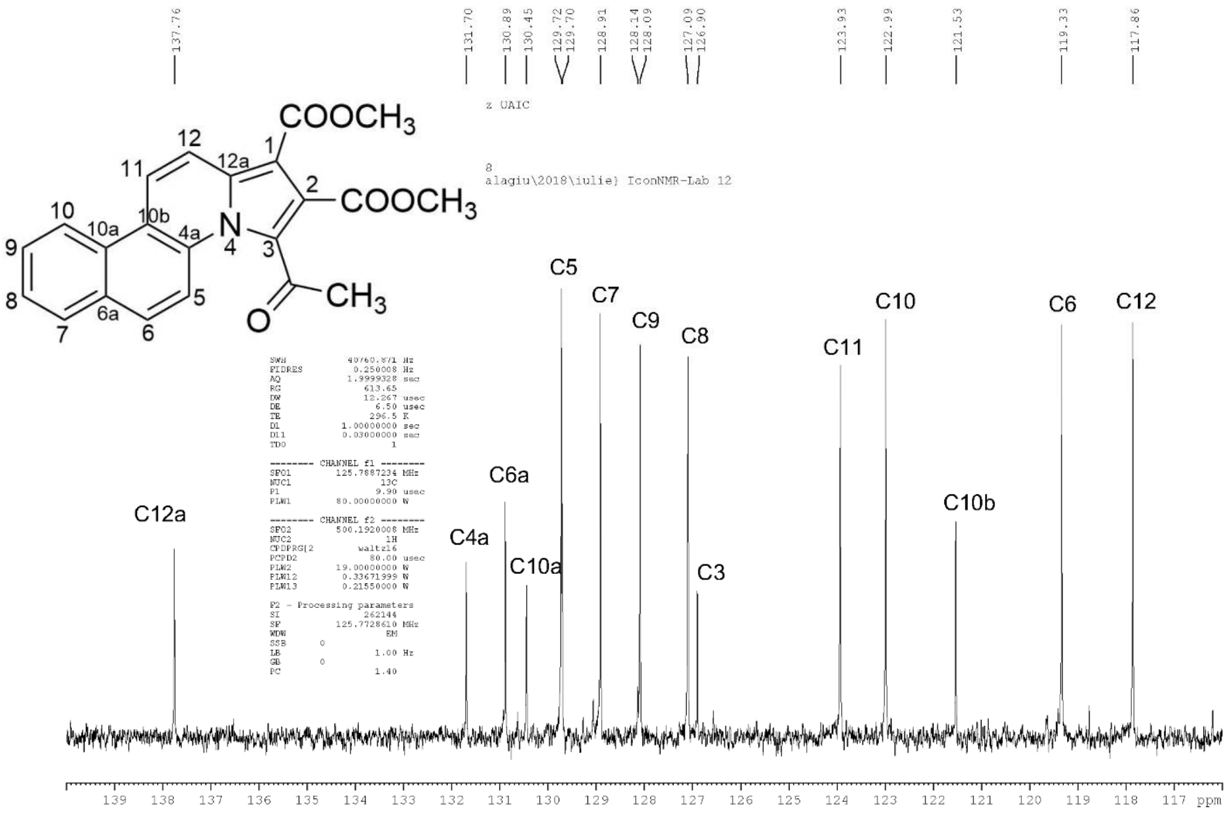

Figure S4c. Detail in the aromatic area of the ^{13}C NMR spectrum of the compound **7a.**

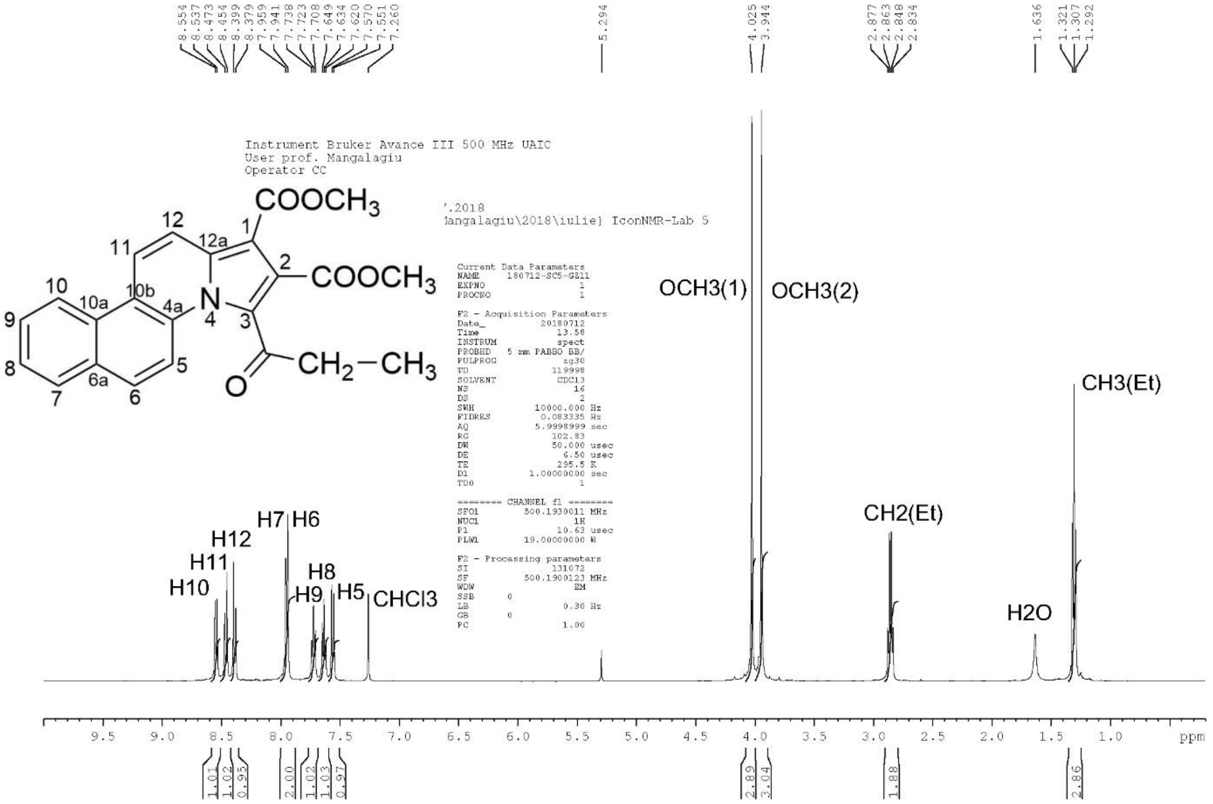

Figure S5a. ^{1}H NMR spectrum of the compound **7b.**

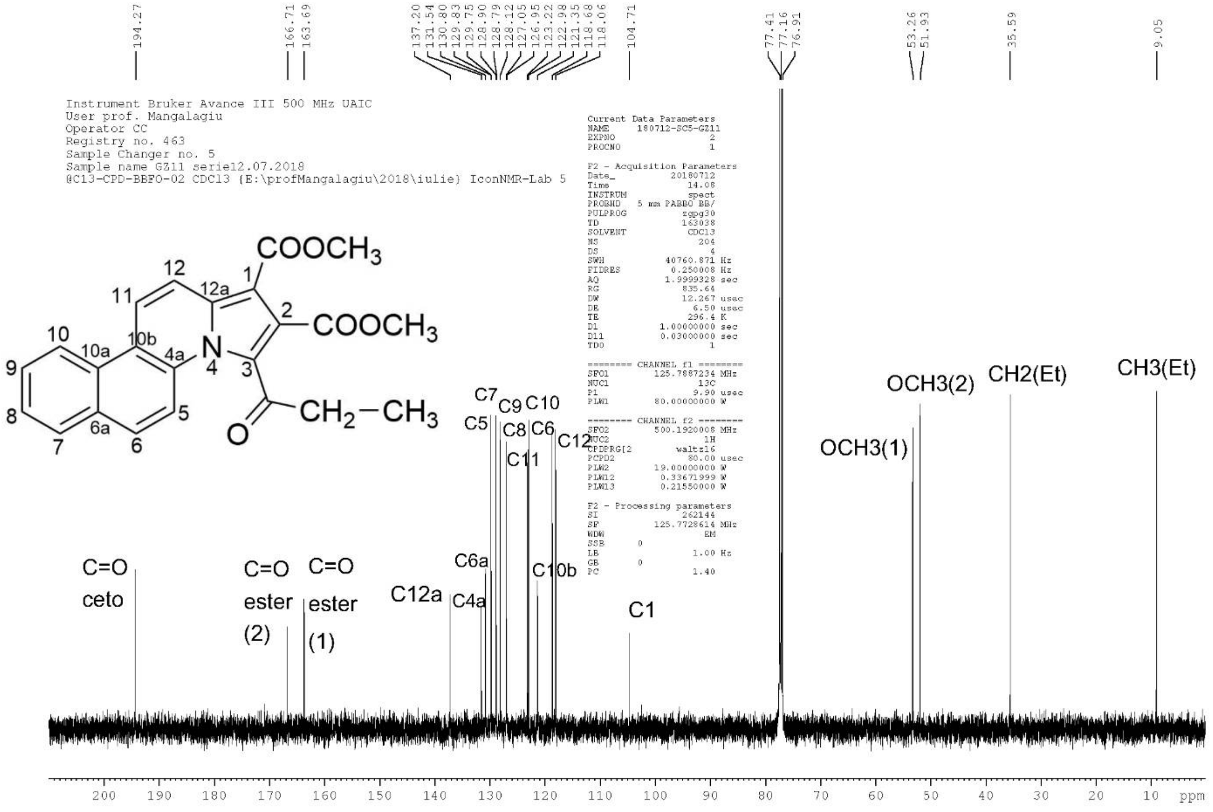

Figure S5b. ^{13}C NMR spectrum of the compound **7b.**

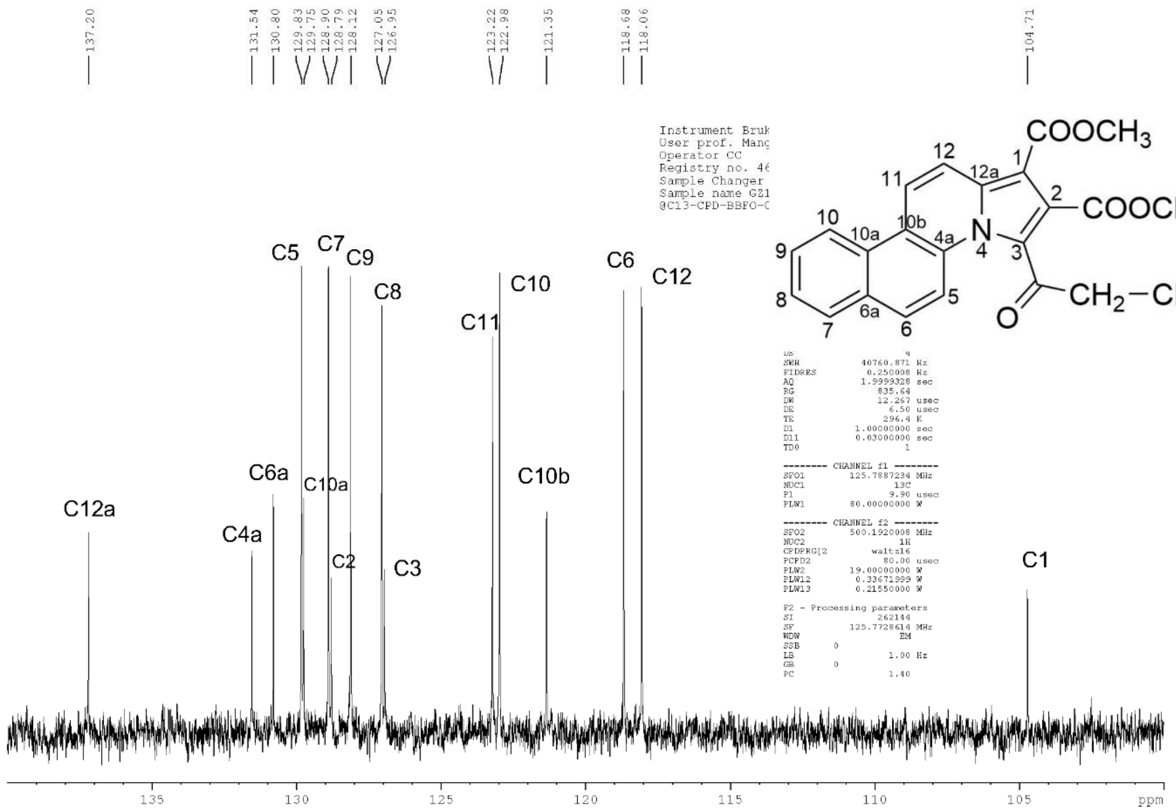

Figure S5c. Detail in the aromatic area of the ^{13}C NMR spectrum of the compound **7b.**

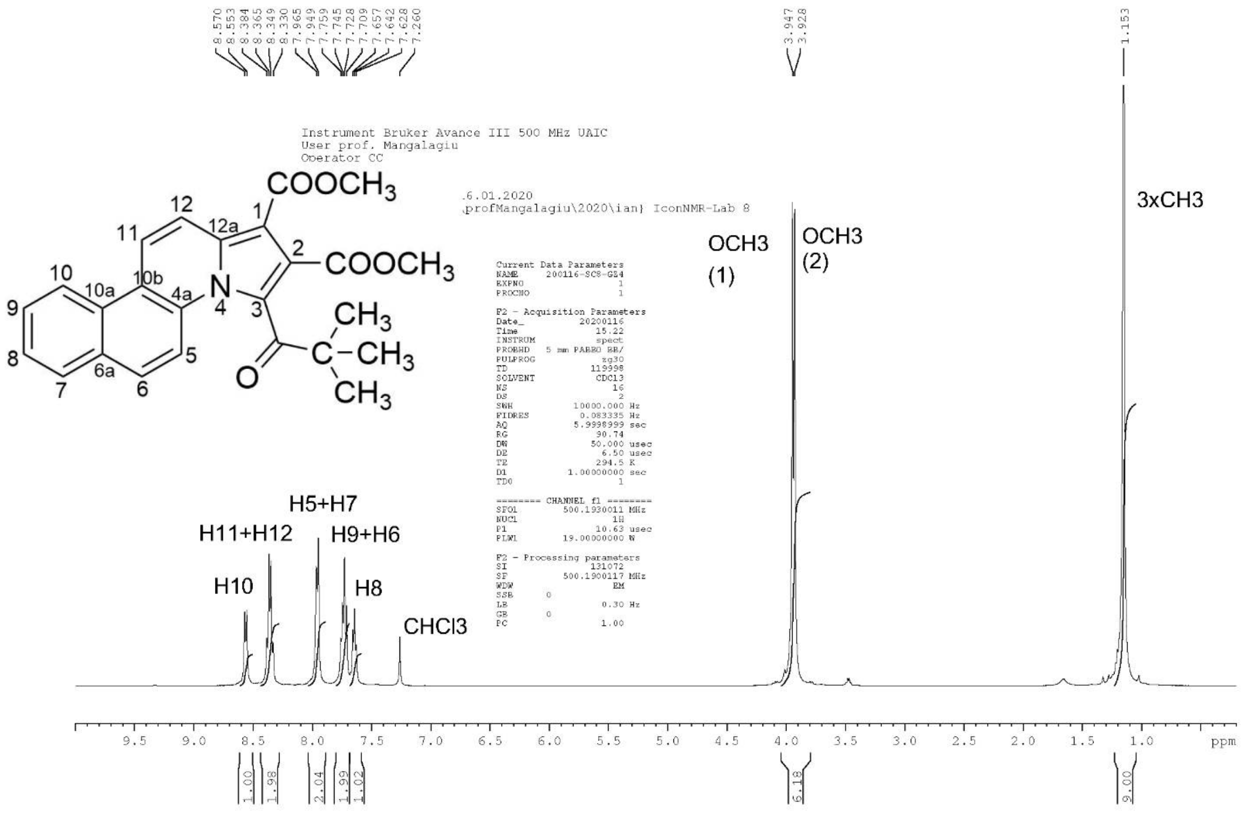

Figure S6a. ^{1}H NMR spectrum of the compound **7c.**

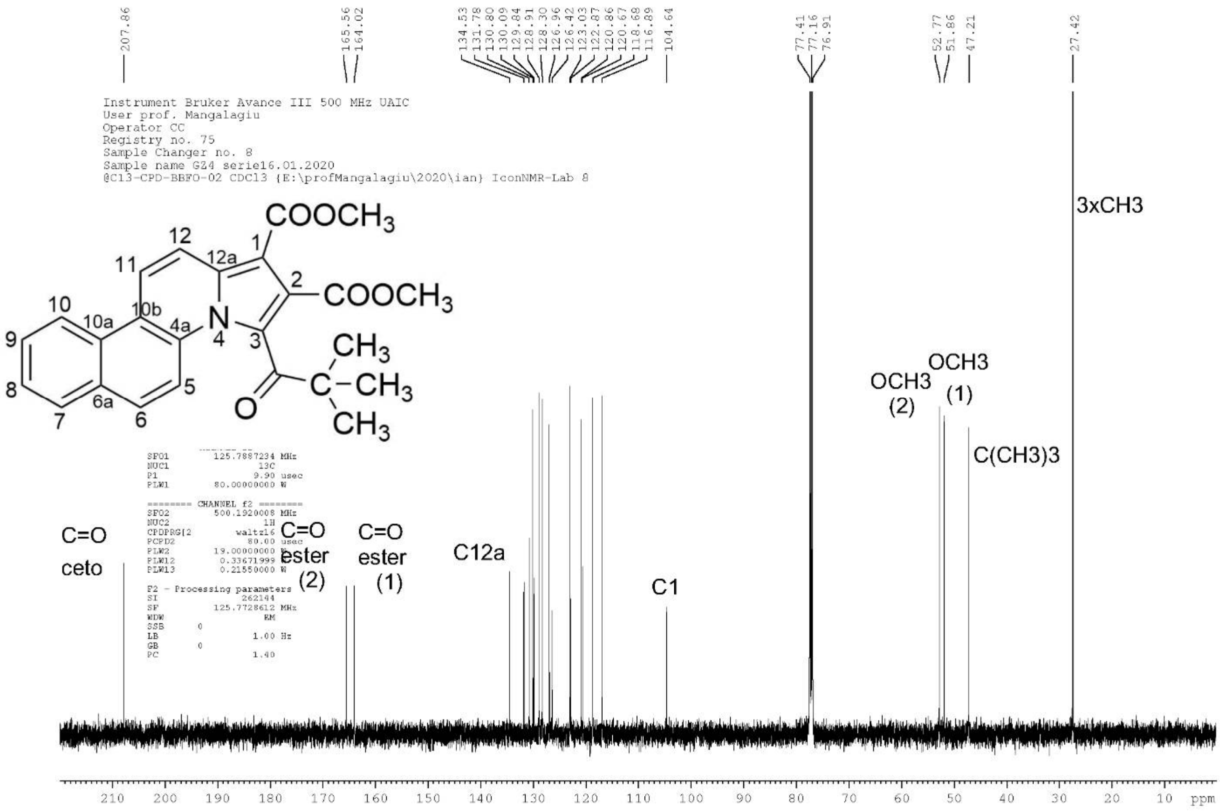

Figure S6b. ^{13}C NMR spectrum of the compound **7c.**

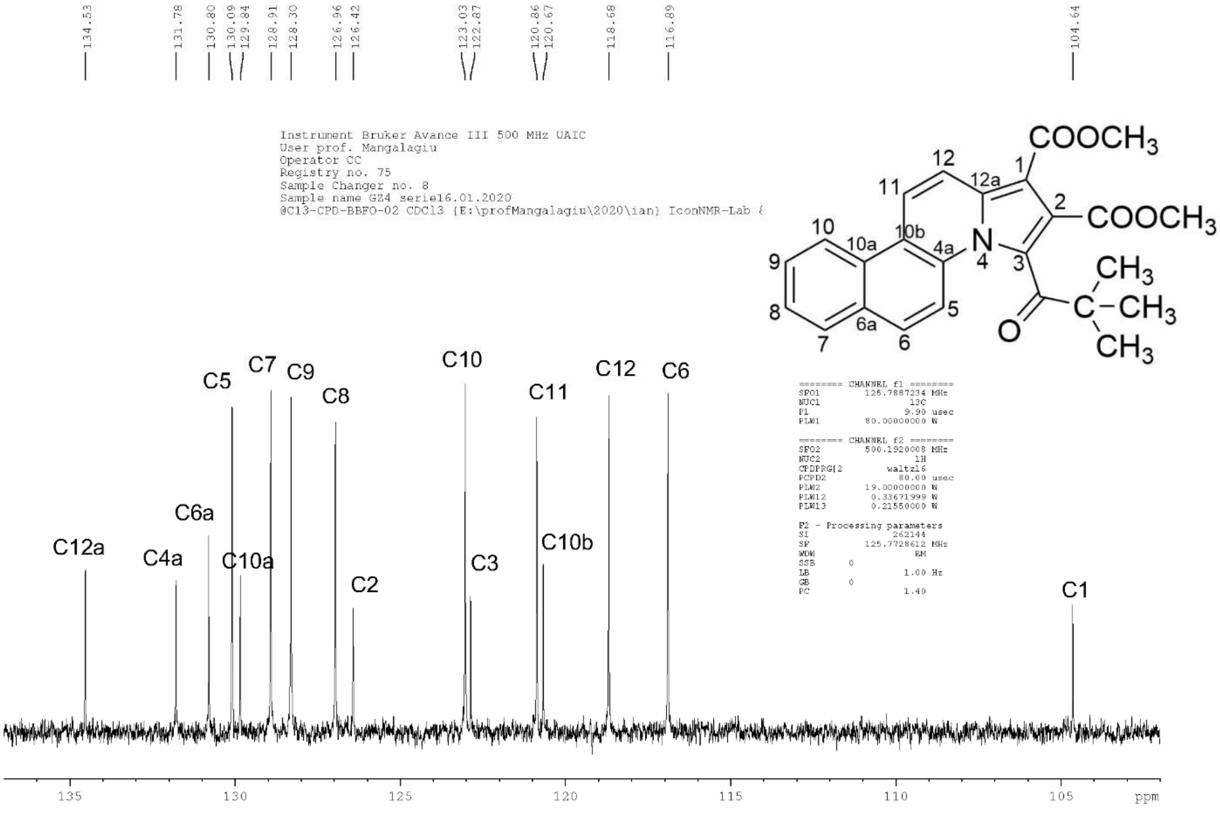

Figure S6c. Detail in the aromatic area of the ^{13}C NMR spectrum of the compound **7c.**

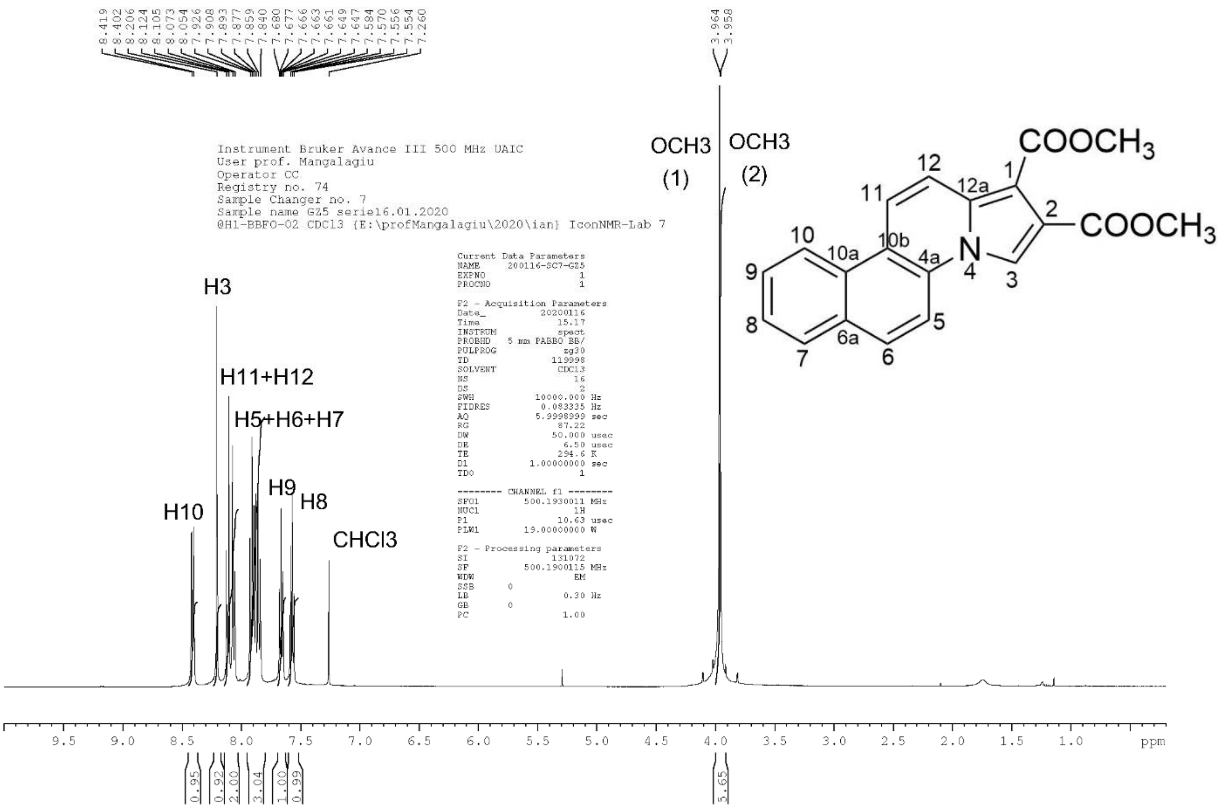

Figure S7a. ^{1}H NMR spectrum of the compound **8c.**

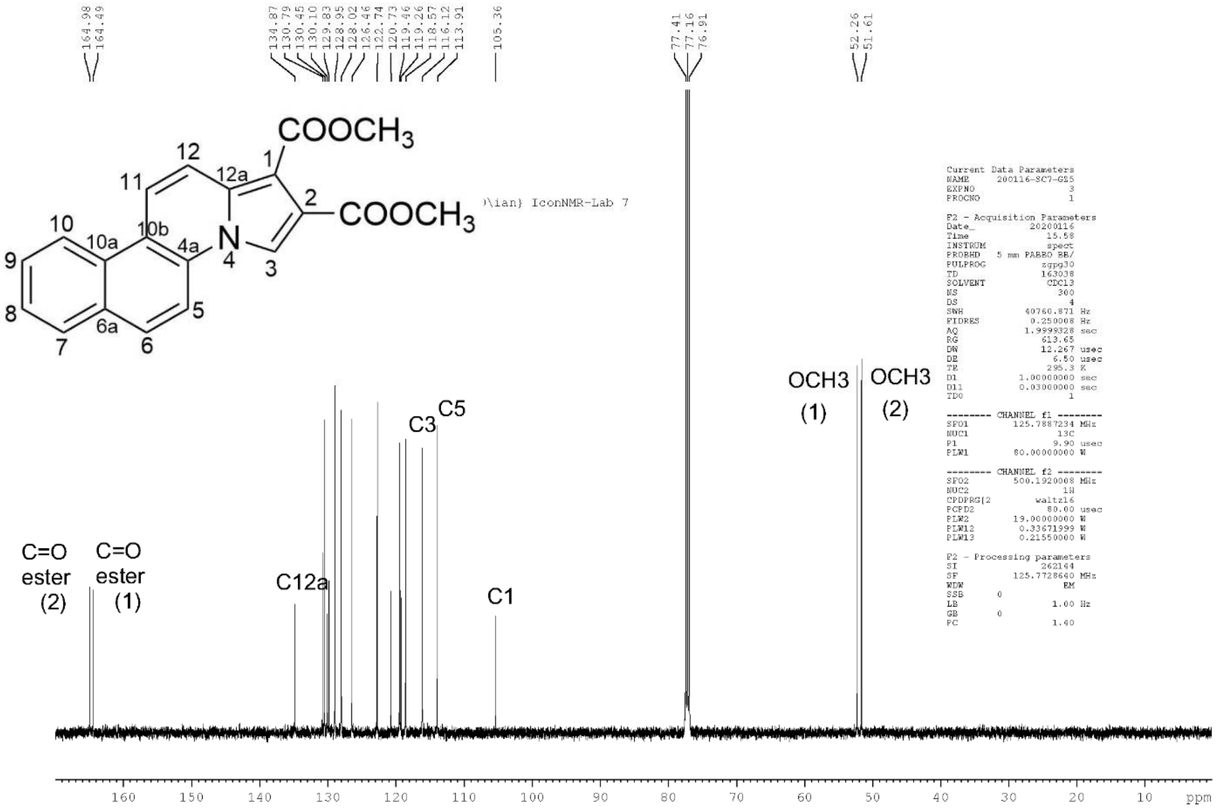

Figure S7b. ^{13}C NMR spectrum of the compound **8c.**

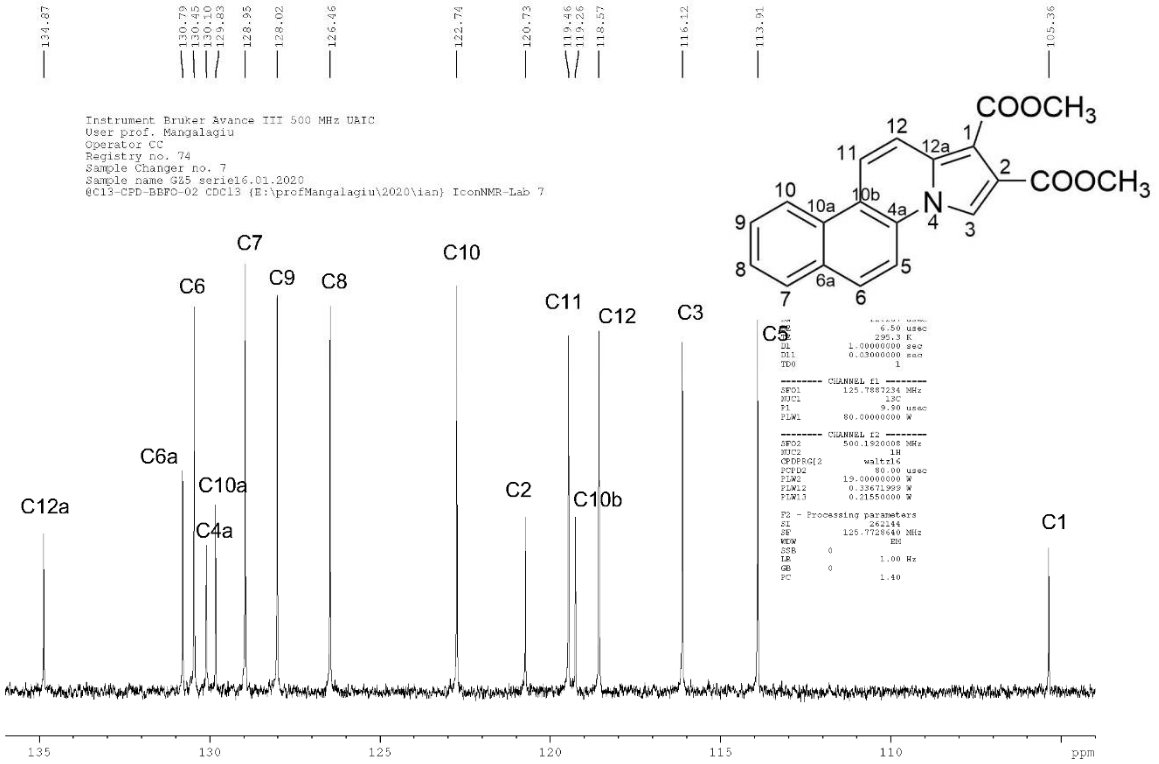

Figure S7c. Detail in the aromatic area of the ^{13}C NMR spectrum of the compound **8c.**

2. IR spectra of the obtained compounds.

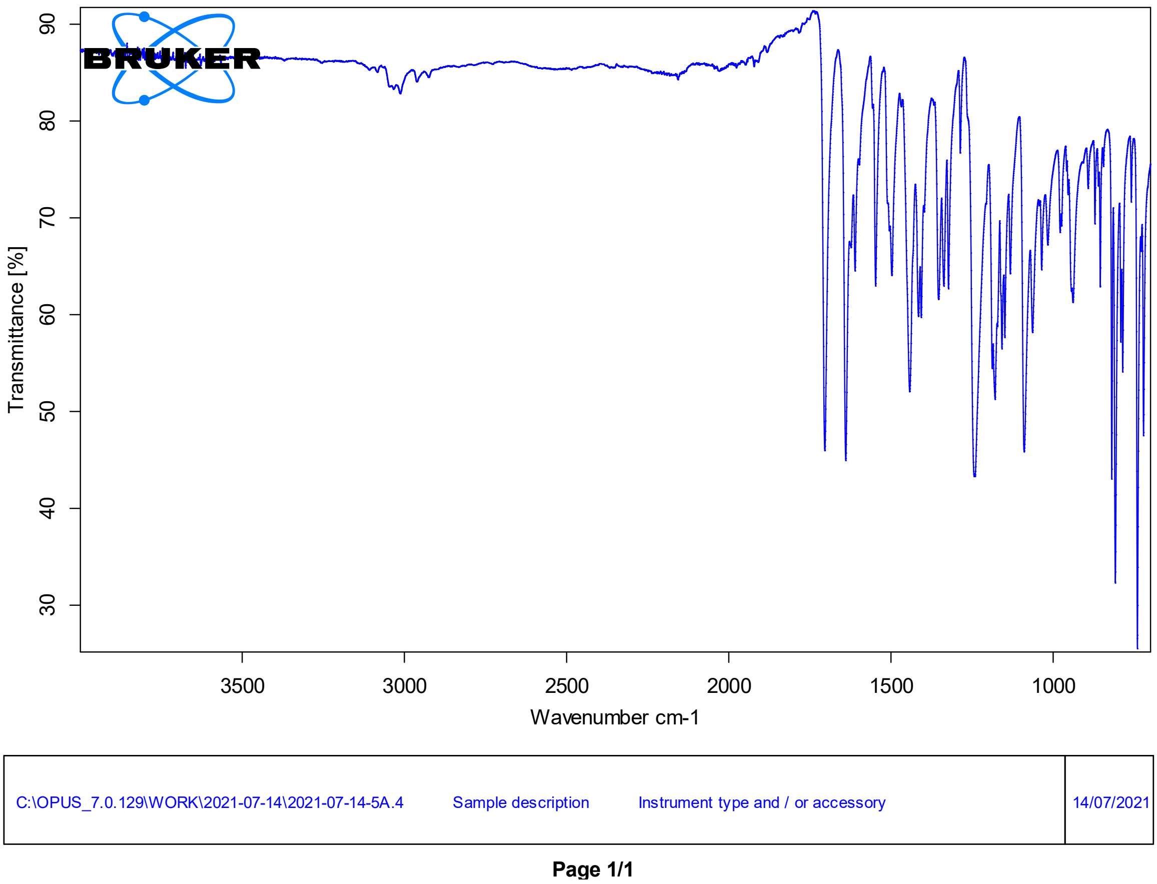

Figure S8. IR spectrum of the compound **5a.**

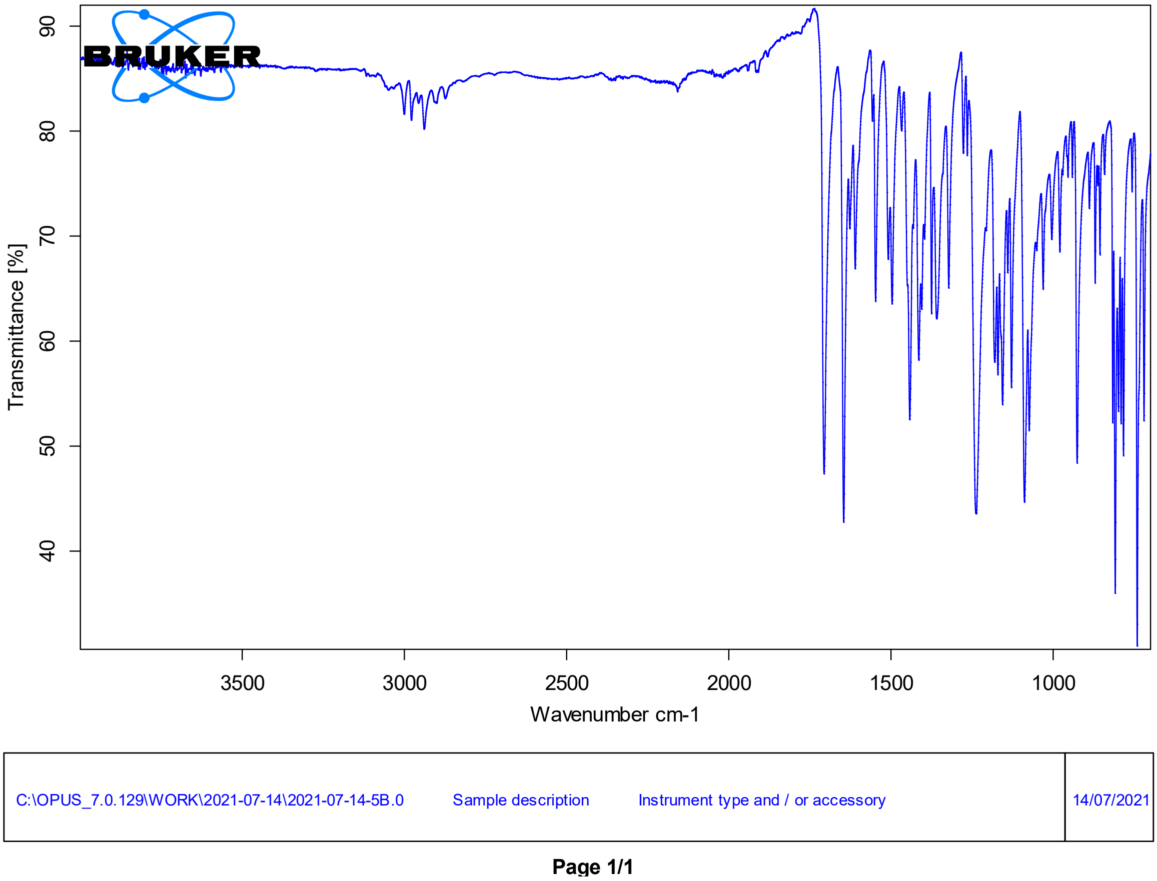

Figure S9. IR spectrum of the compound **5b.**

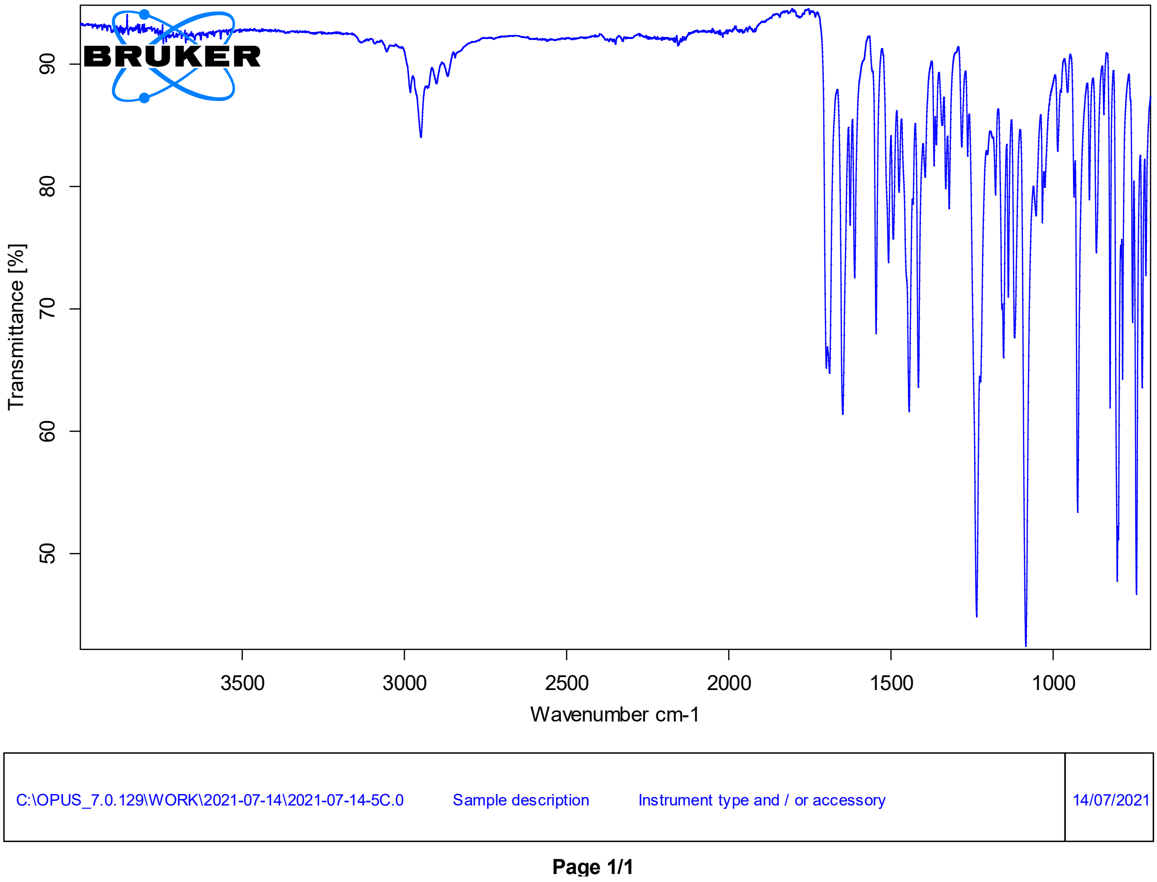

Figure S10. IR spectrum of the compound **5c.**

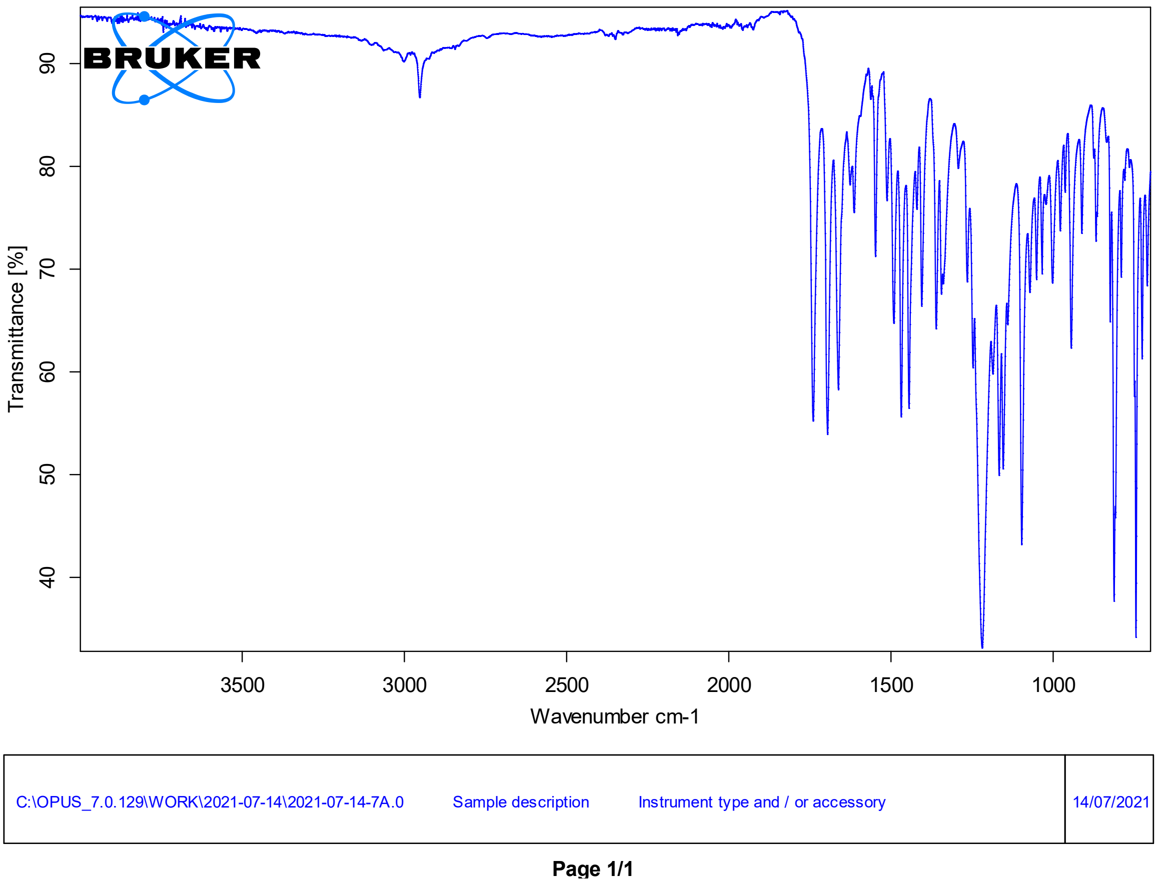

Figure S11. IR spectrum of the compound **7a.**

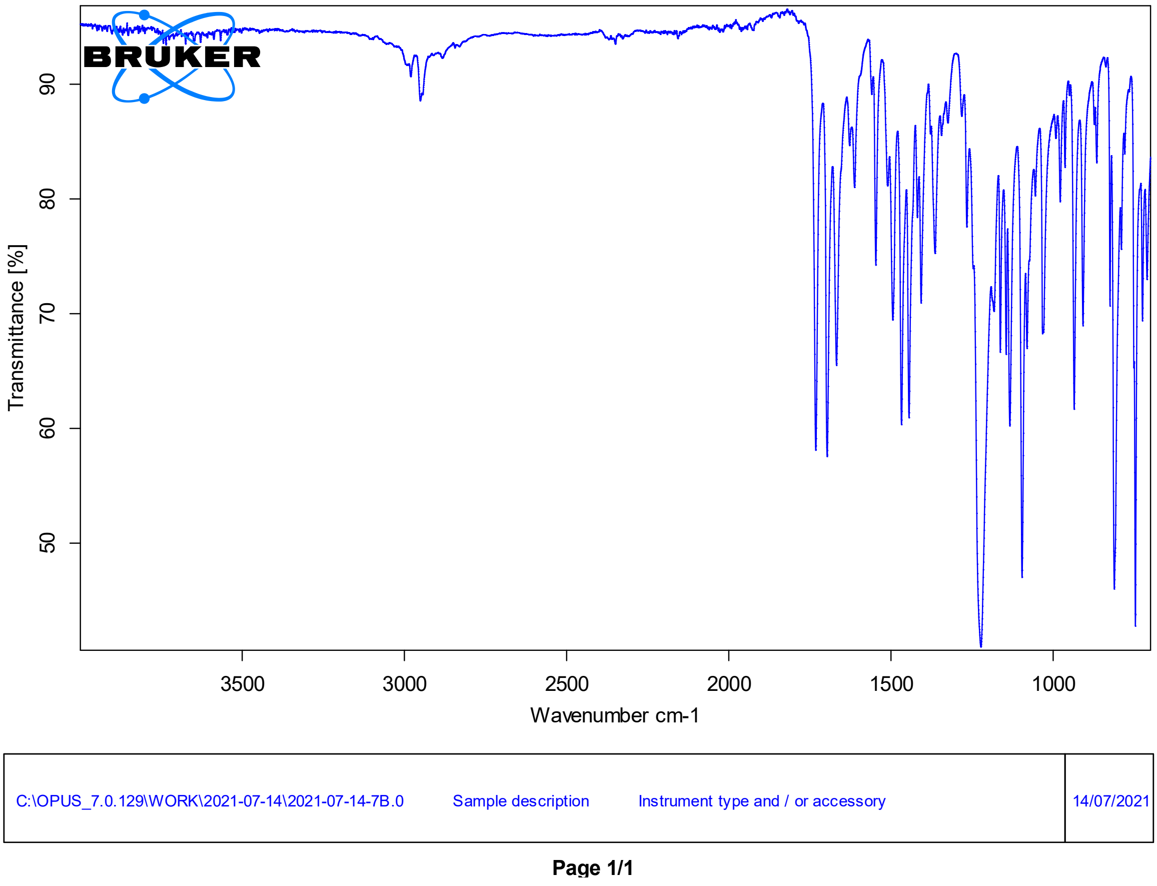

Figure S12. IR spectrum of the compound **7b.**

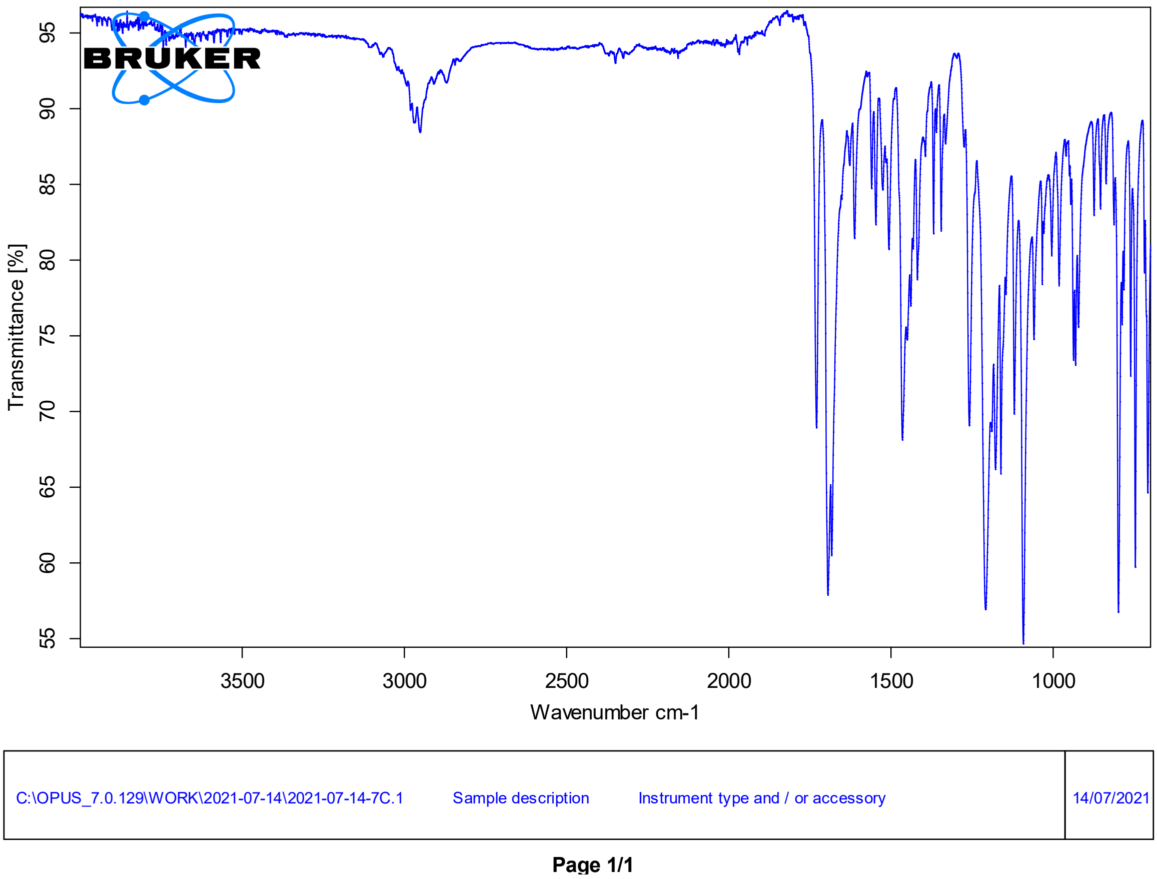

Figure S13. IR spectrum of the compound **7c.**

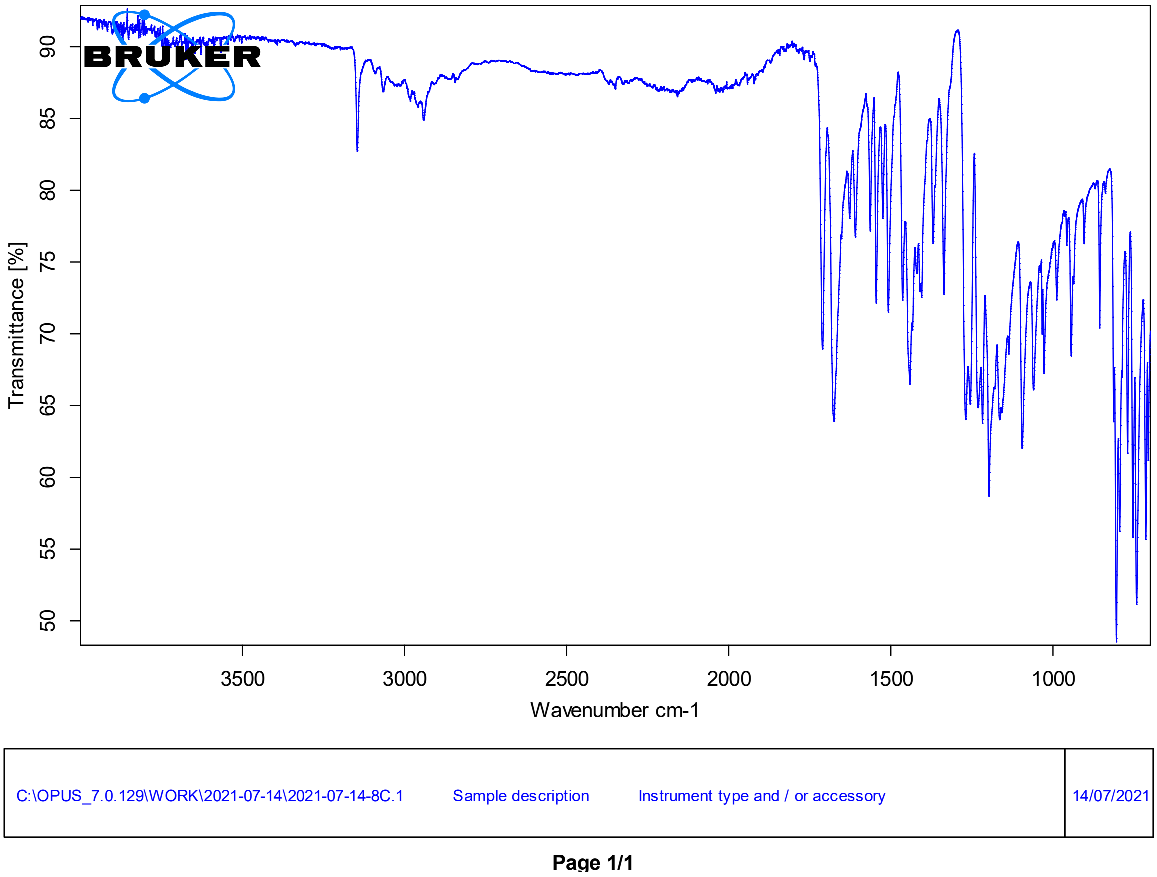

Figure S14. IR spectrum of the compound **8c.**

3. ^{1}H-NMR studies on compound 7a at room temperature.

NMR sample preparation:

10 mg of dimethyl 3-acetylbenzo[f]pyrrolo[1,2-a]quinoline-1,2-dicarboxylate (**7a**) were dissolved in 1 mL $CDCl_3$ and inserted in a 5 mm diameter NMR tube and sealed with a polypropylene cap. A ^{1}H-NMR spectrum was quickly recorded than the sample was stored at room temperature. After 1 day, 2 days and 5 days respectively a new ^{1}H-NMR spectrum was recorded for the sample.

The ^{1}H-NMR spectra are presented in the S15 Fig

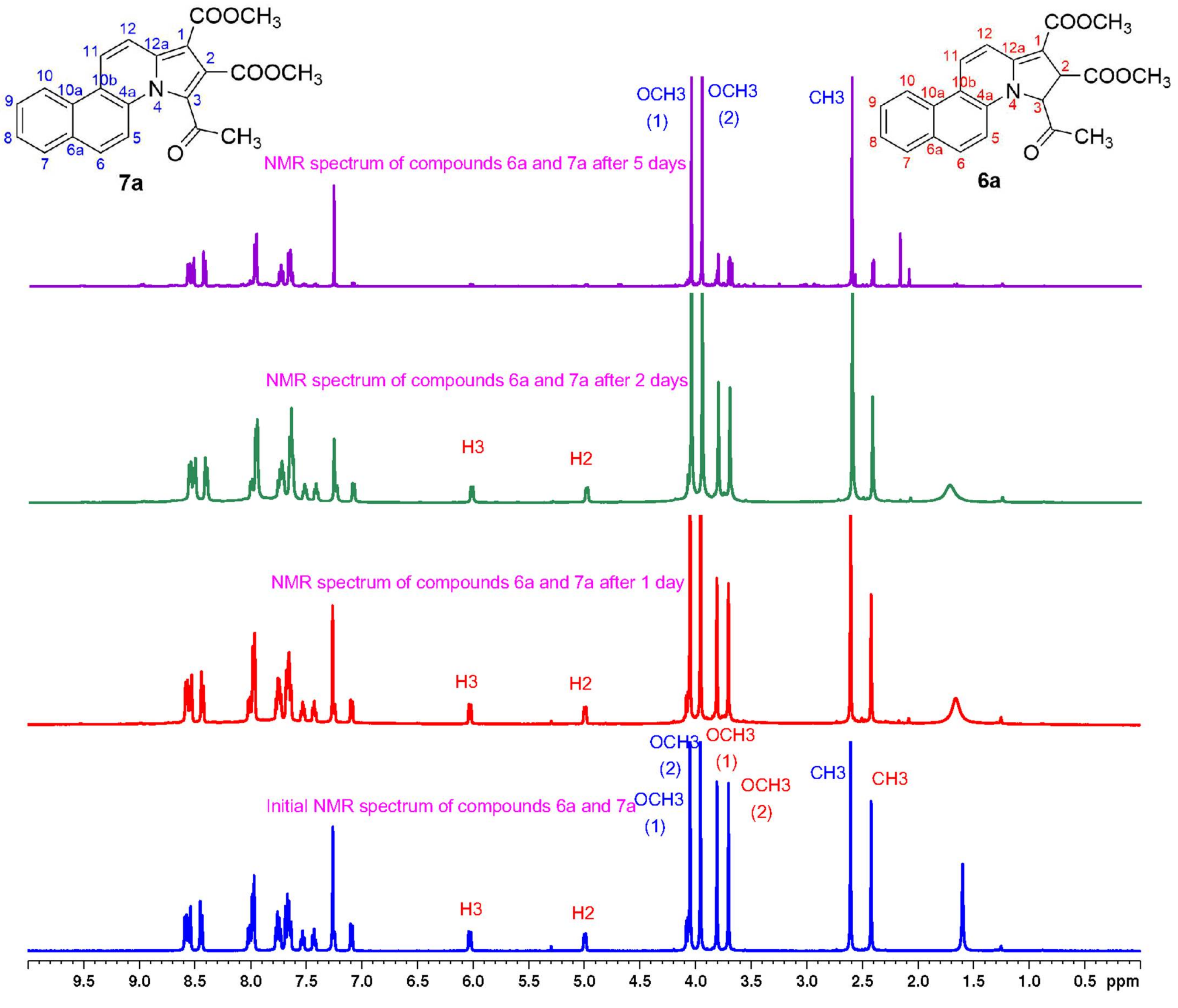

Figure S15. Time evolution of the ^{1}H-NMR spectrum of dimethyl 3-acetylbenzo[f]pyrrolo[1,2-a]quinoline-1,2-dicarboxylate (**7a**).

4. UV spectra of the obtained compounds

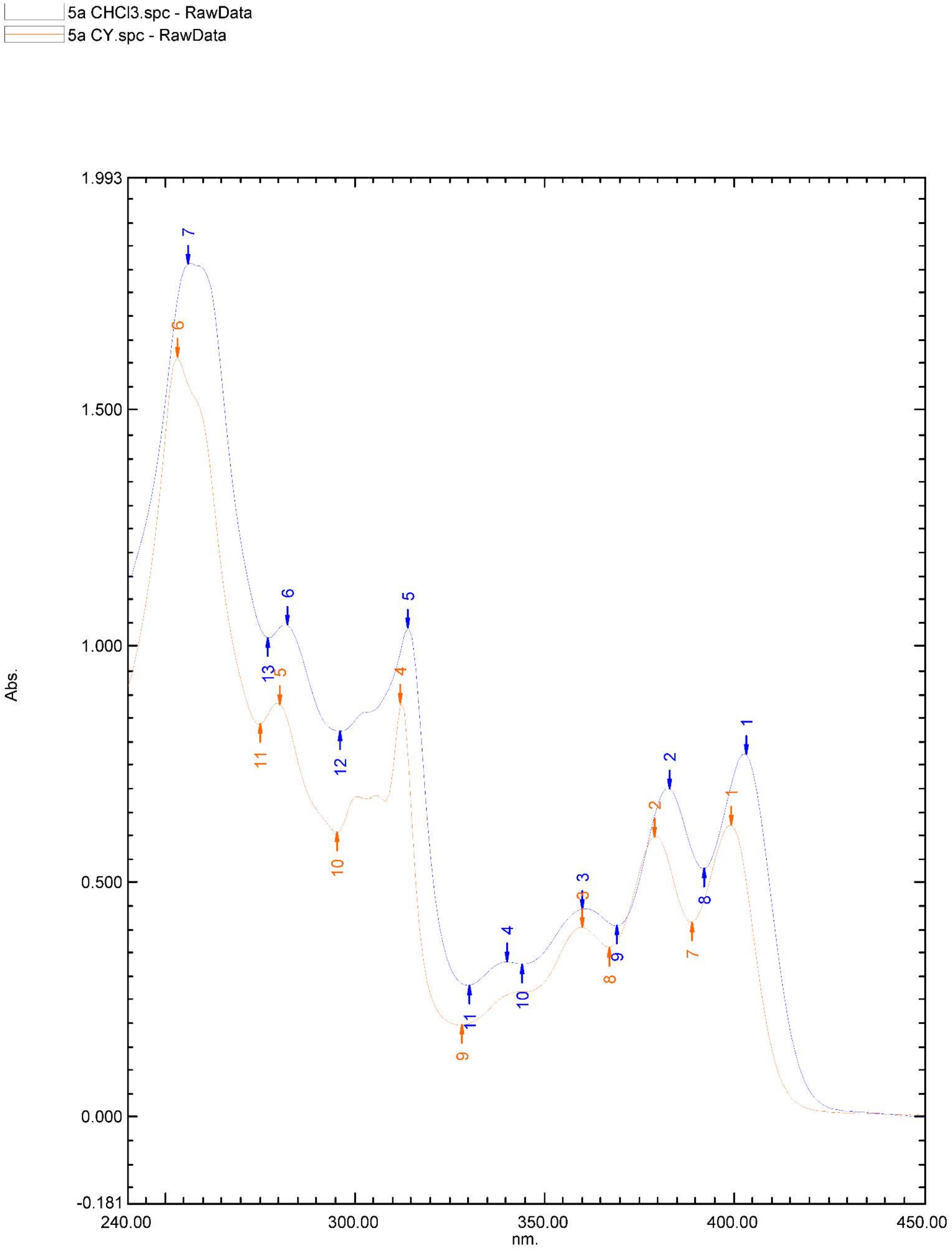

Figure S16. Overlayed UV-Vis spectra of the compound **5a** in trichloromethane and cyclohexane.

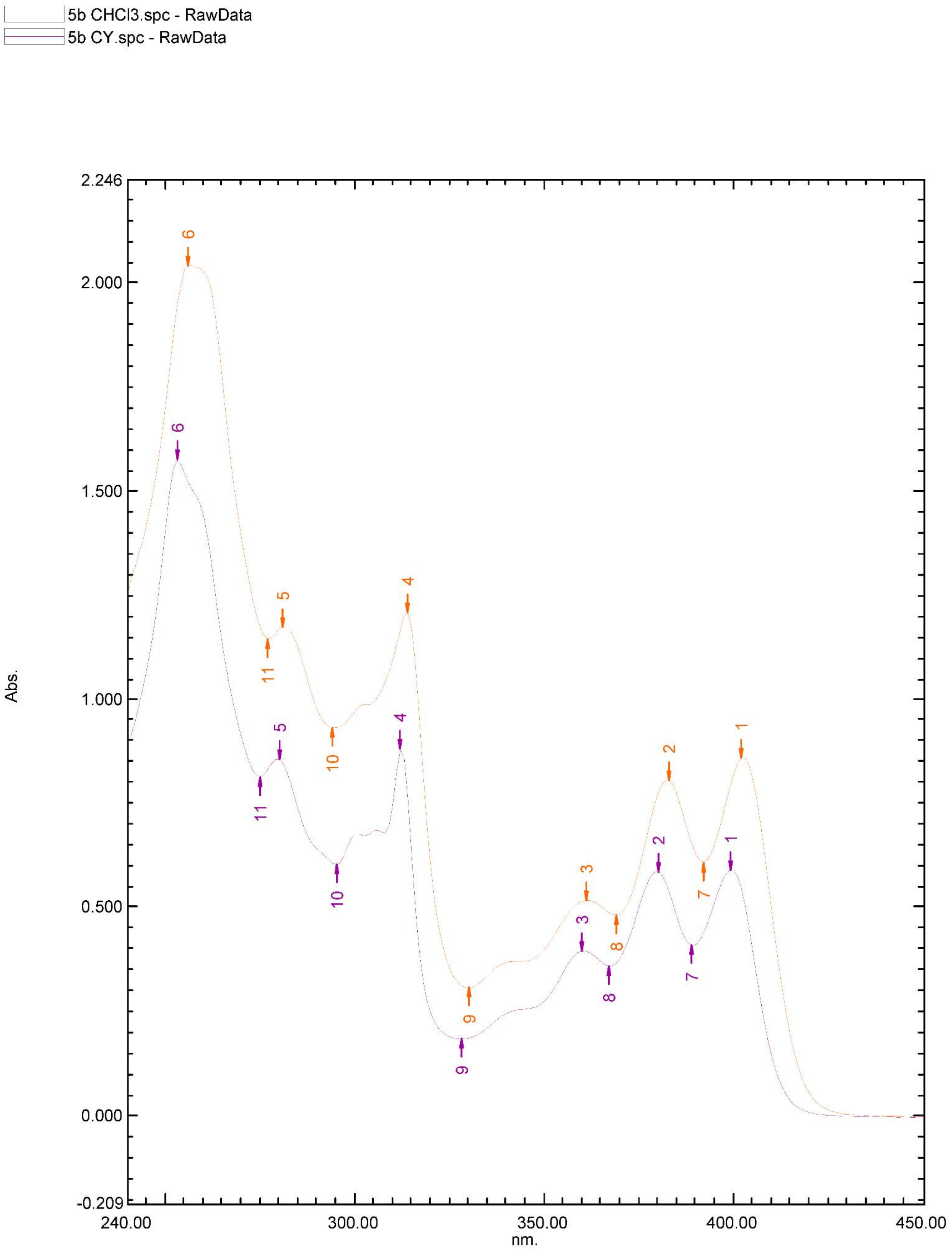

Figure S17. Overlayed UV-Vis spectra of the compound **5b** in trichloromethane and cyclohexane.

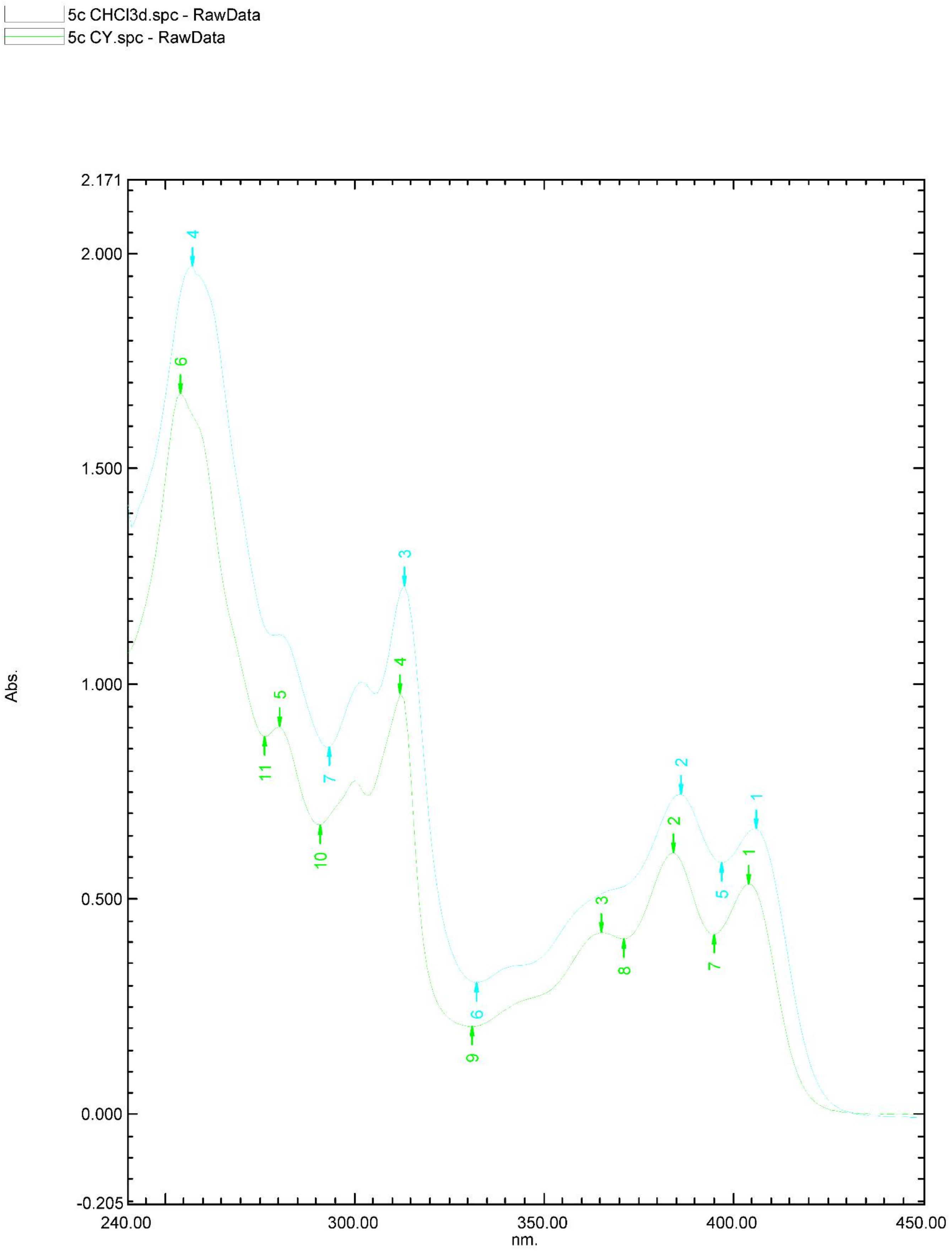

Figure S18. Overlayed UV-Vis spectra of the compound **5c** in trichloromethane and cyclohexane.

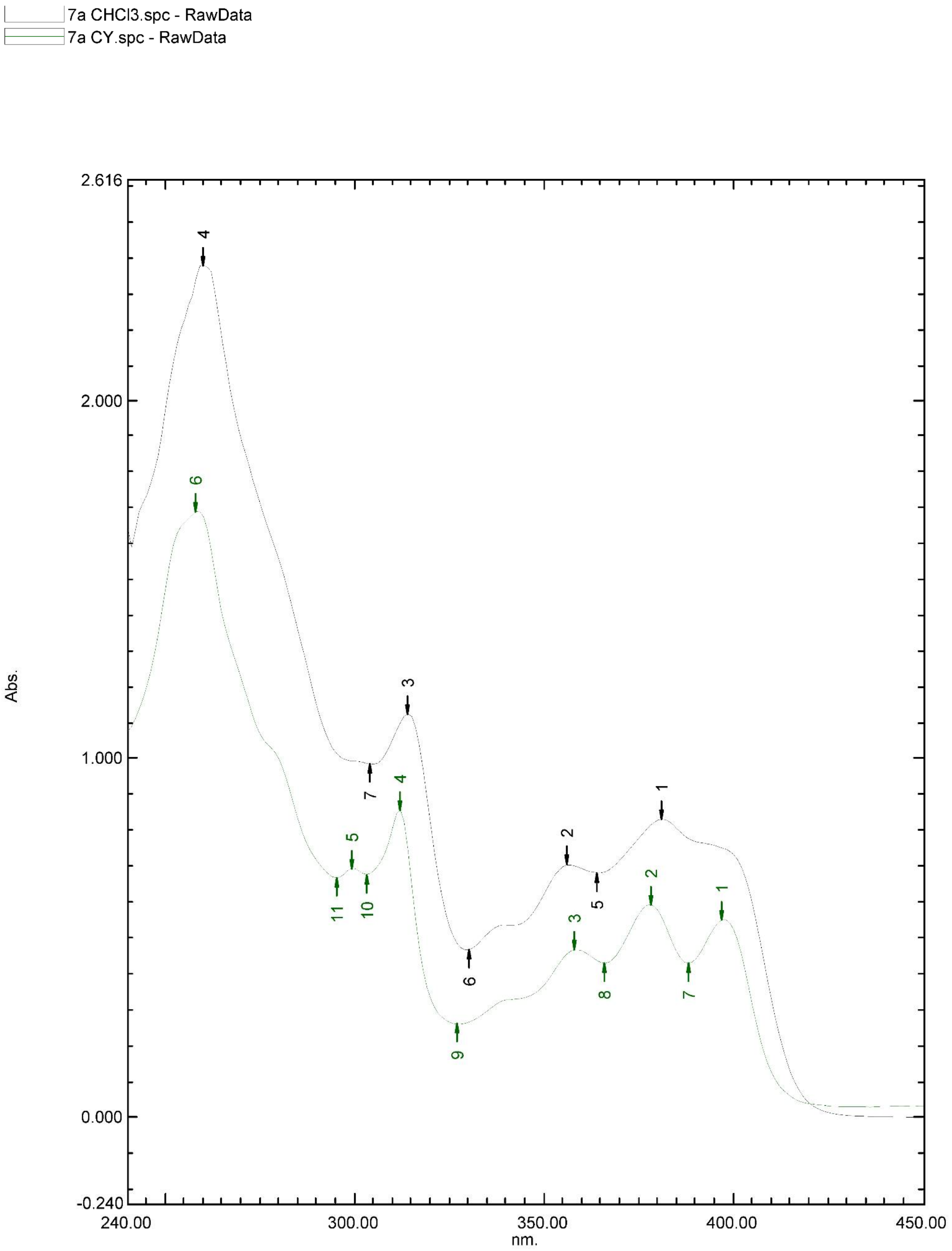

Figure S19. Overlayed UV-Vis spectra of the compound **7a** in trichloromethane and cyclohexane.

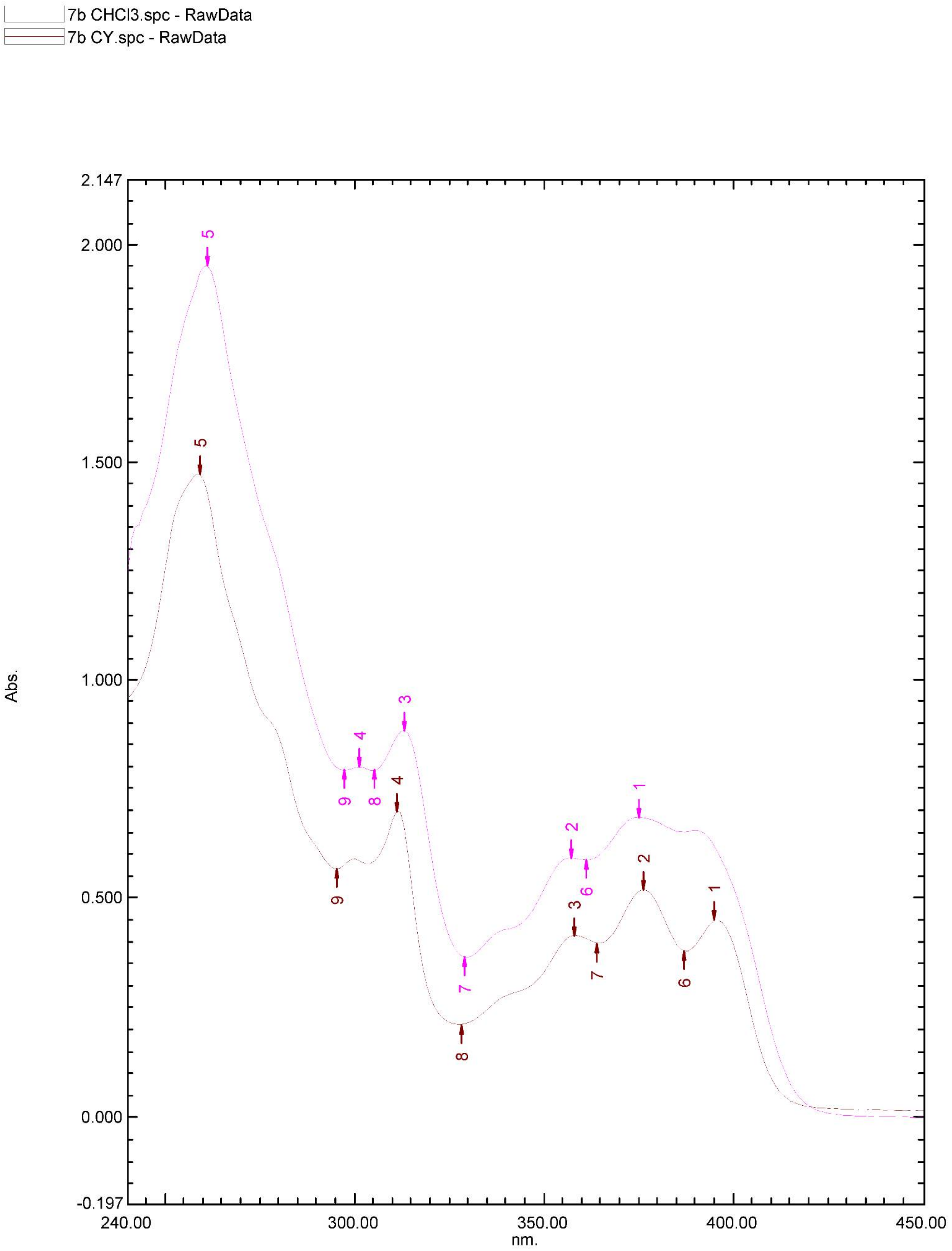

Figure S20. Overlayed UV-Vis spectra of the compound **7b** in trichloromethane and cyclohexane.

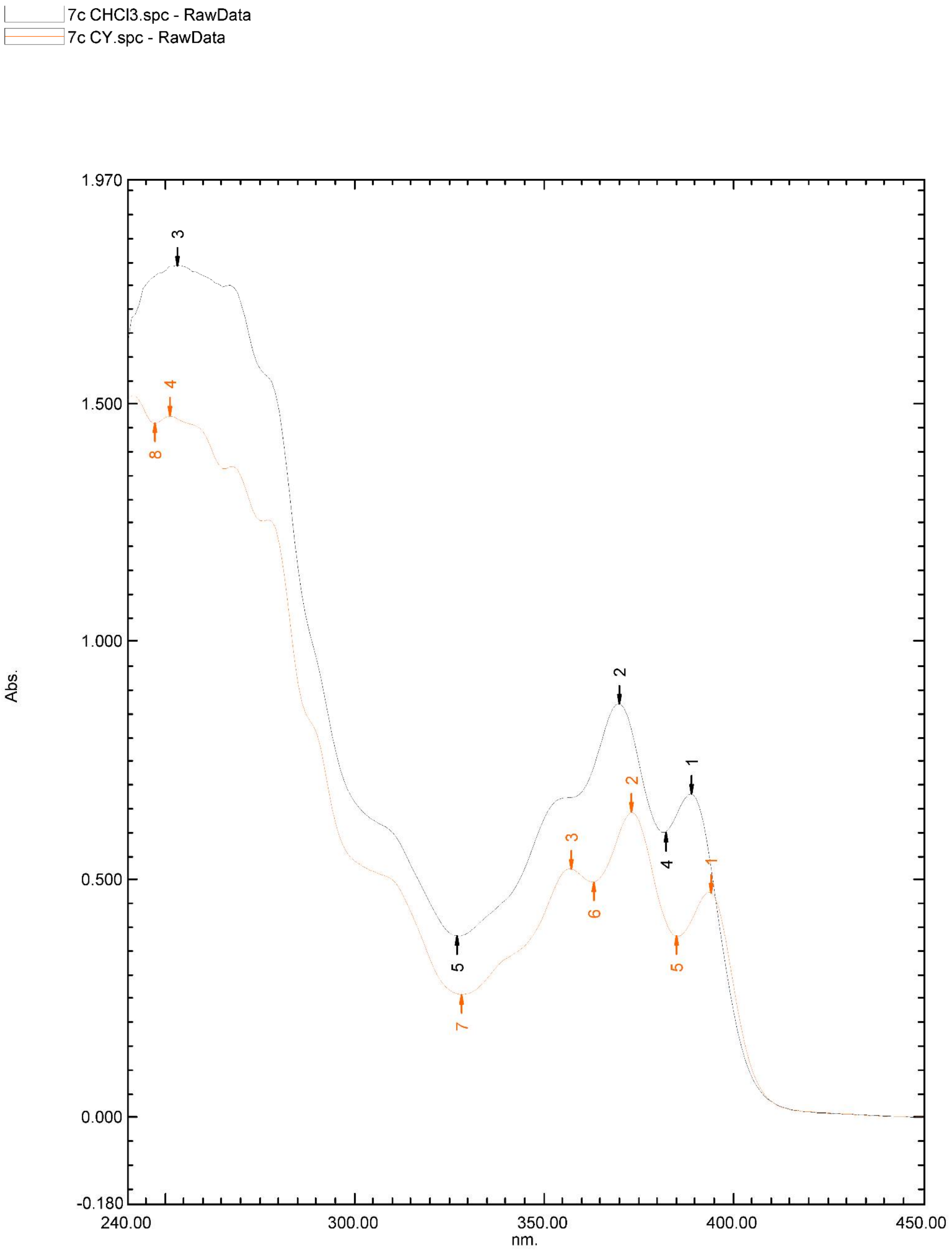

Figure S21. Overlayed UV-Vis spectra of the compound **7c** in trichloromethane and cyclohexane.

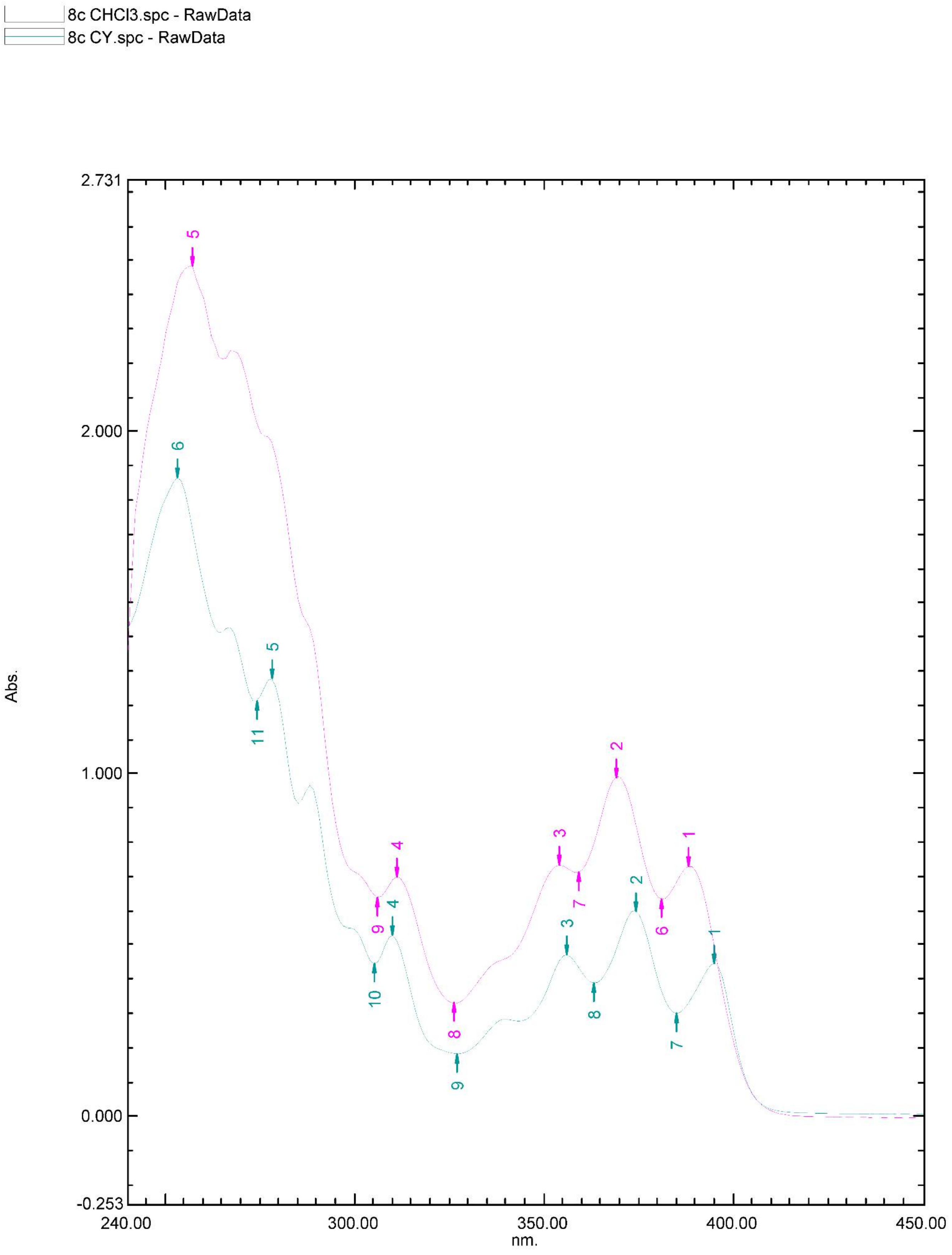

Figure S22. Overlayed UV-Vis spectra of the compound **8c** in trichloromethane and cyclohexane.

5. Emission spectra of the obtained compounds

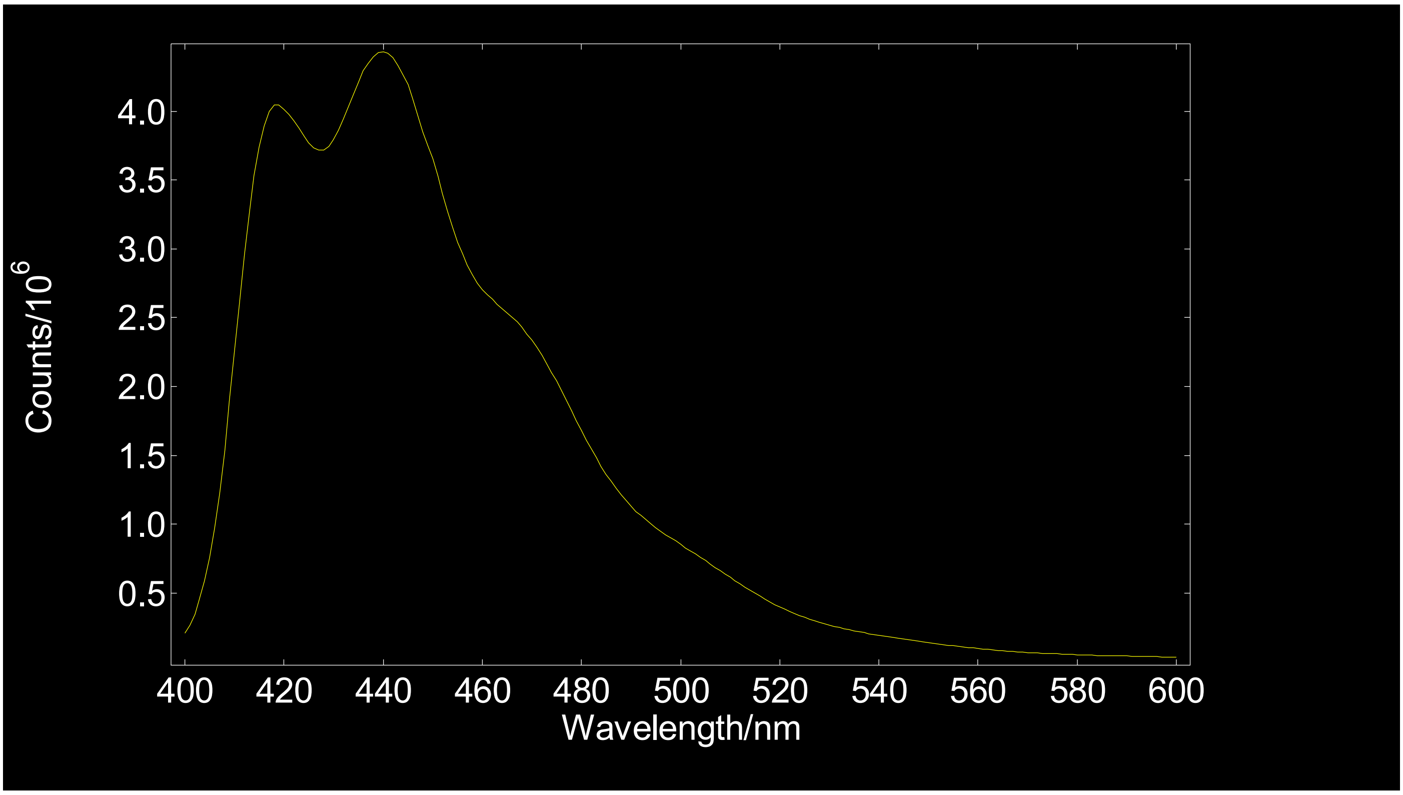

Figure S23. Emission spectrum of the compound **5a** in trichloromethane.

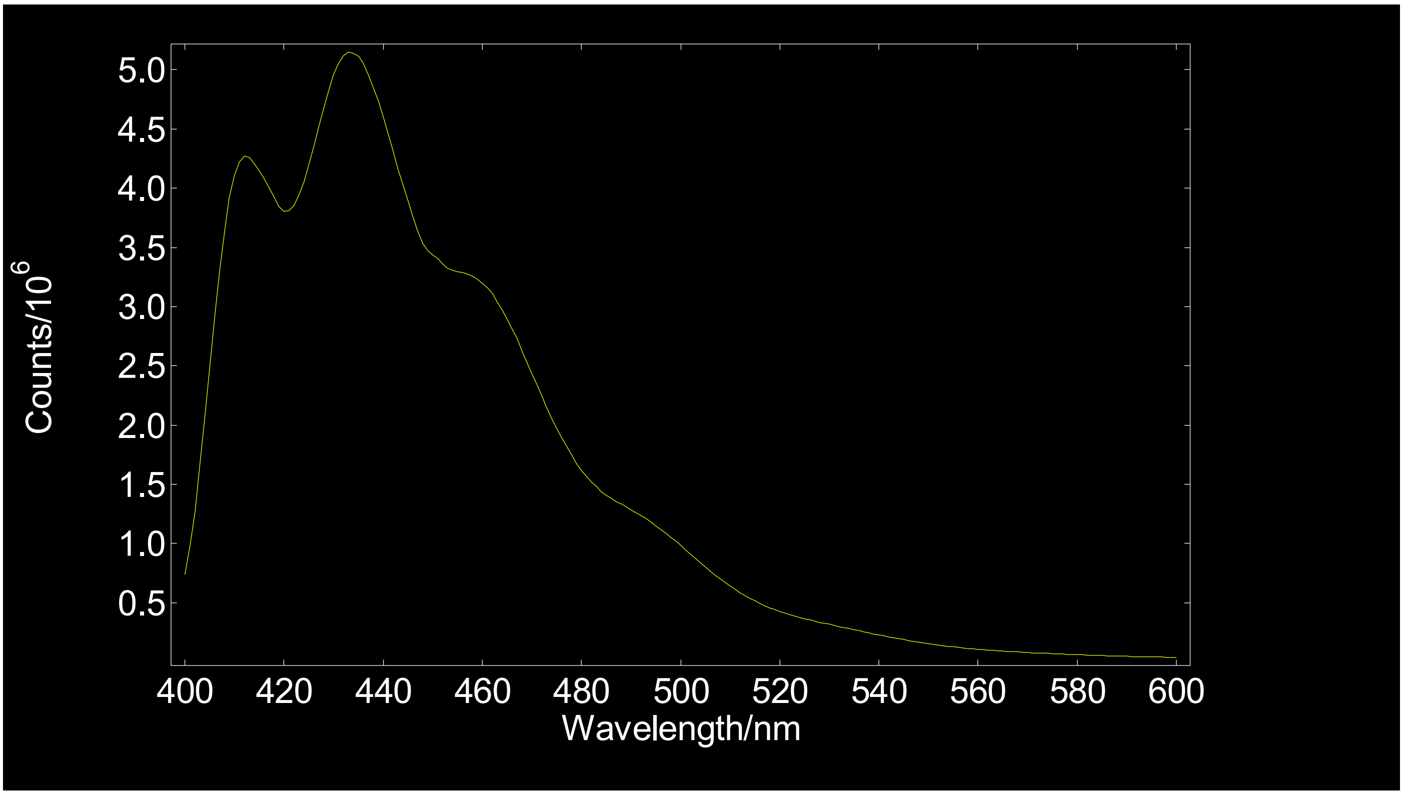

Figure S24. Emission spectrum of the compound **5a** in cyclohexane.

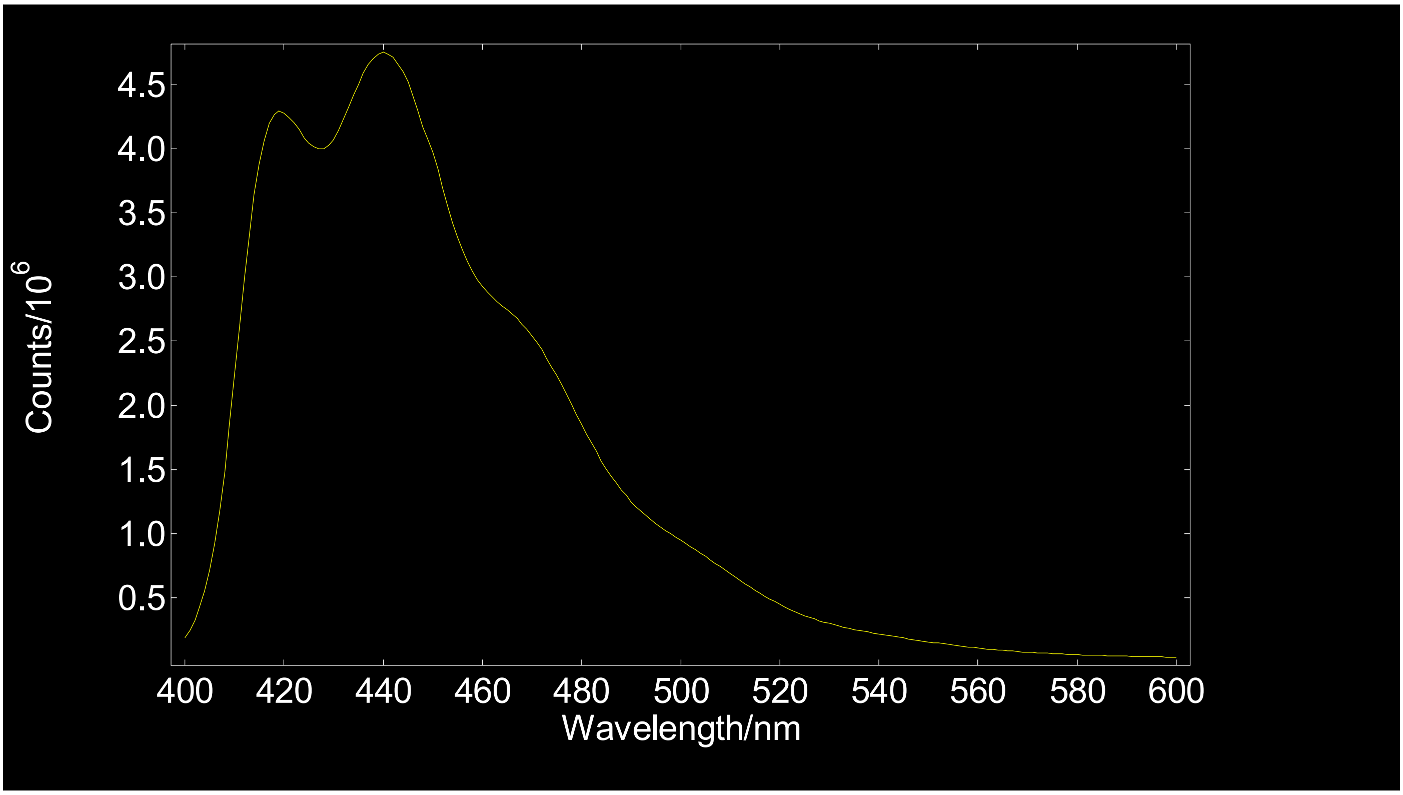

Figure S25. Overlayed UV-Vis spectra of the compound 5b in trichloromethane and cyclohexane.

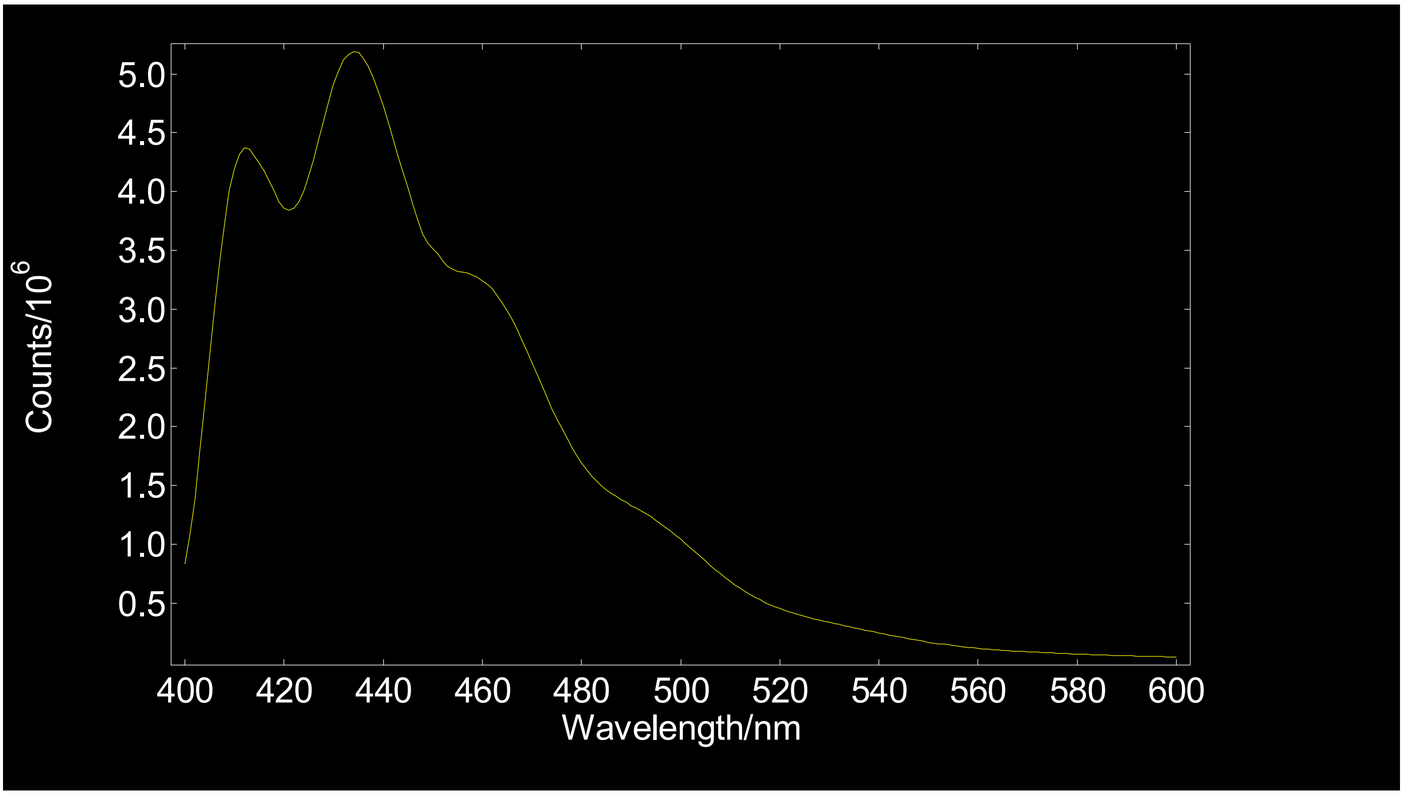

Figure S26. Emission spectrum of the compound **5b** in cyclohexane.

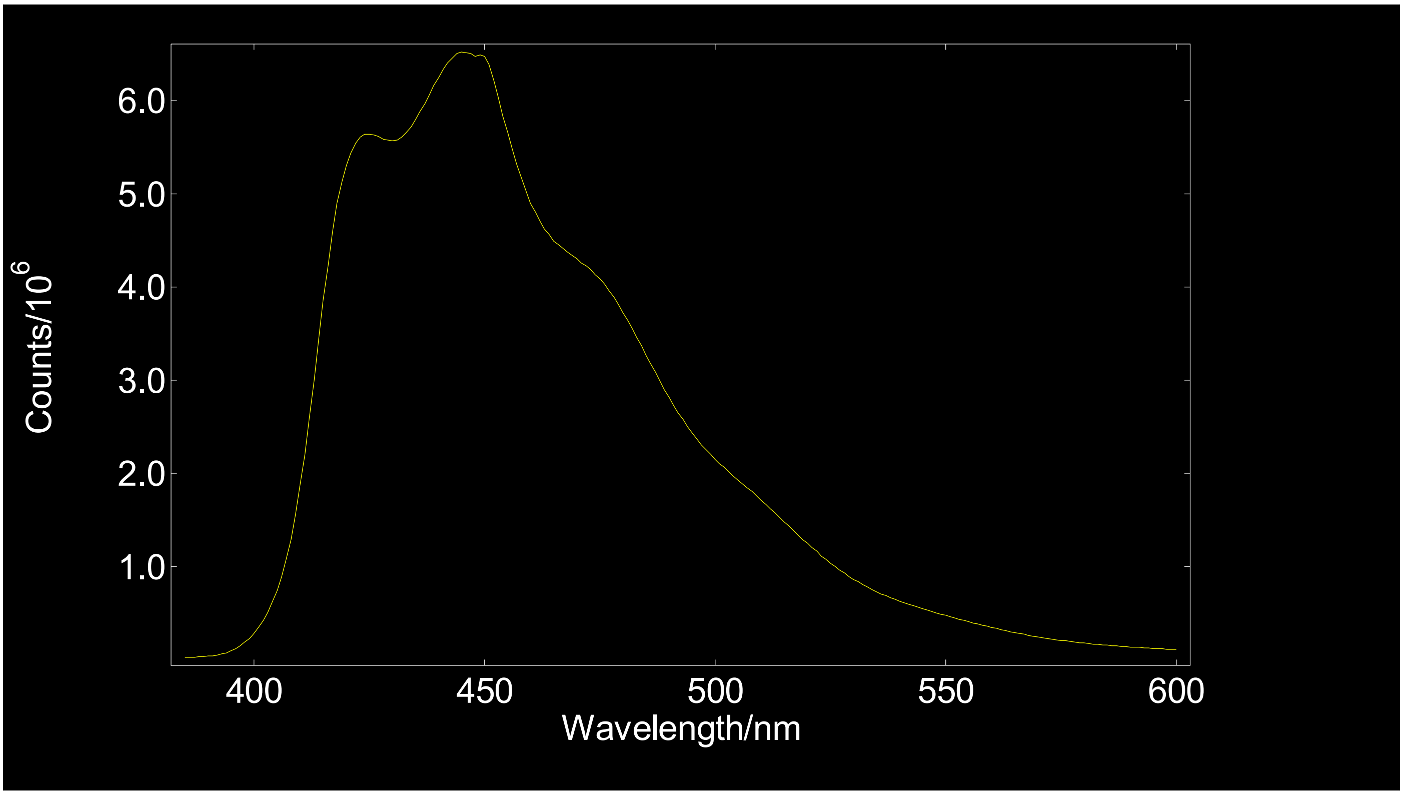

Figure S27. Emission spectrum of the compound **5c** in trichloromethane.

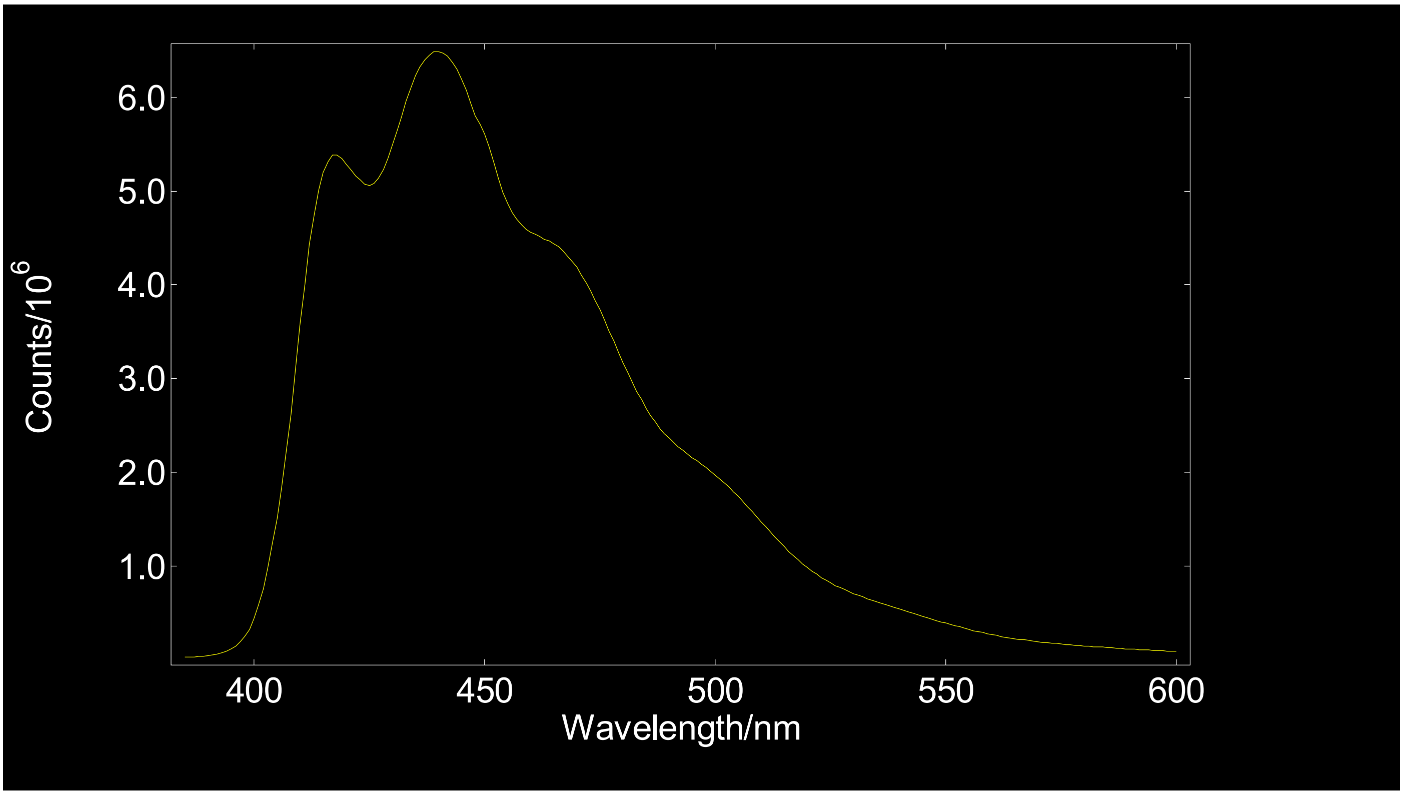

Figure S28. Emission spectrum of the compound **5c** in cyclohexane.

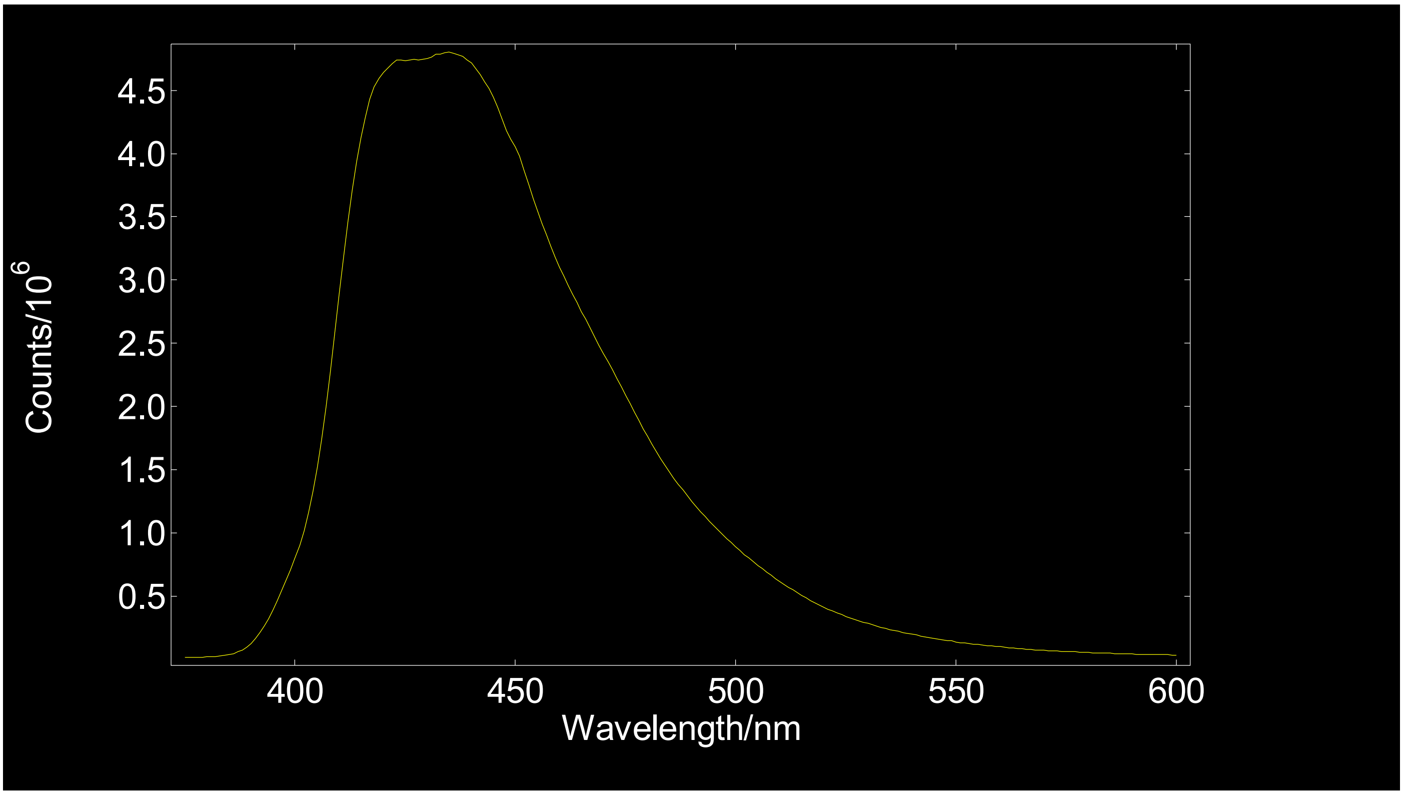

Figure S29. Emission spectrum of the compound **7a** in trichloromethane.

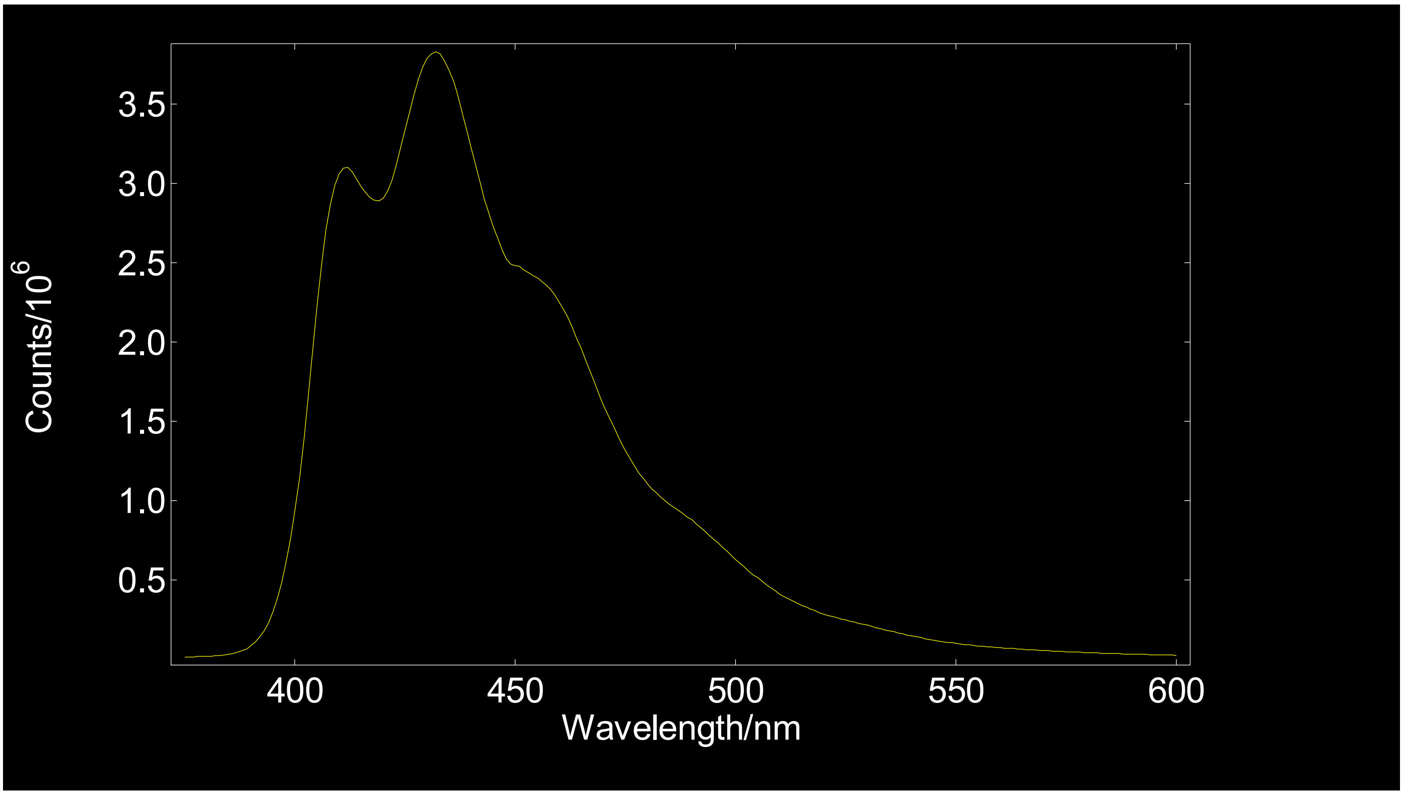

Figure S30. Emission spectrum of the compound **7a** in cyclohexane.

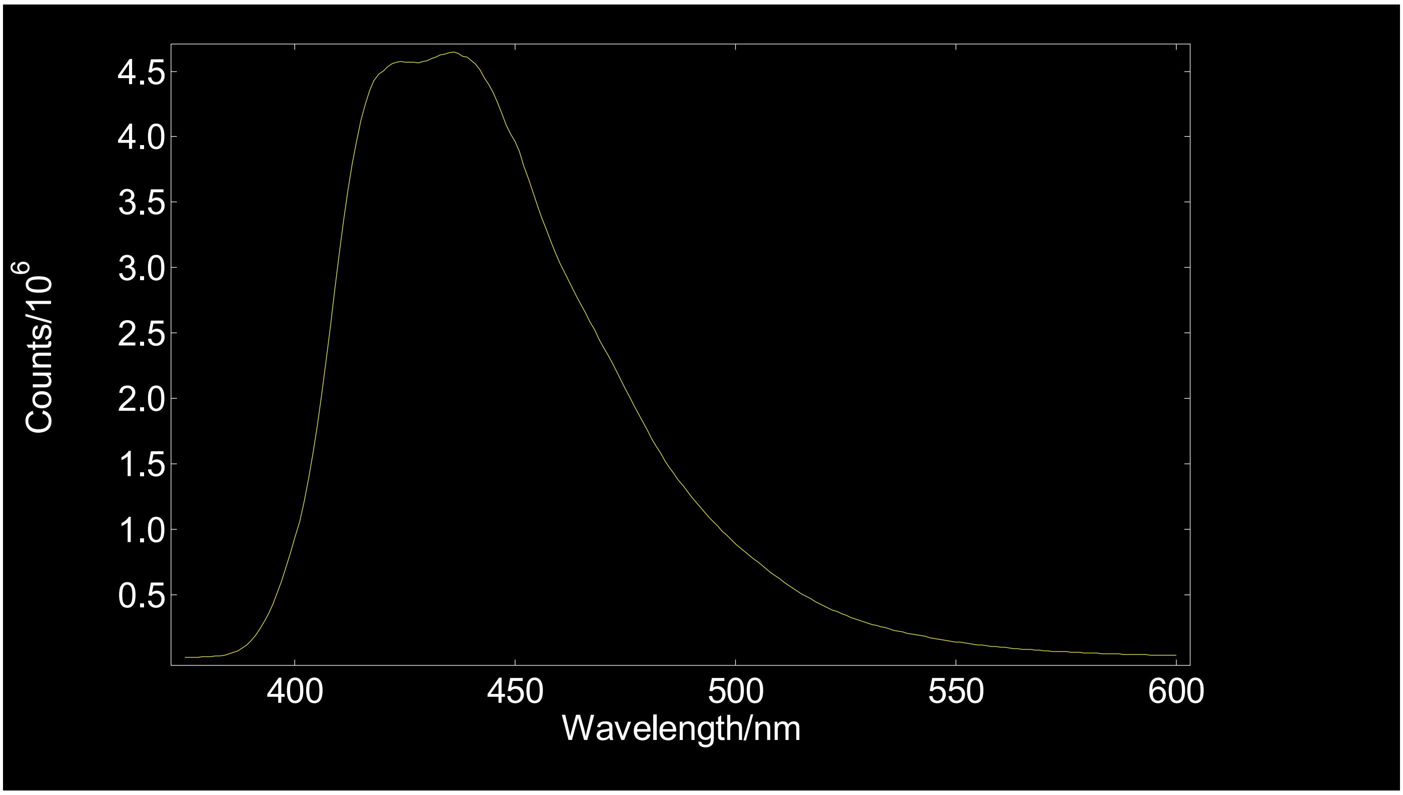

Figure S31. Emission spectrum of the compound **7b** in trichloromethane.

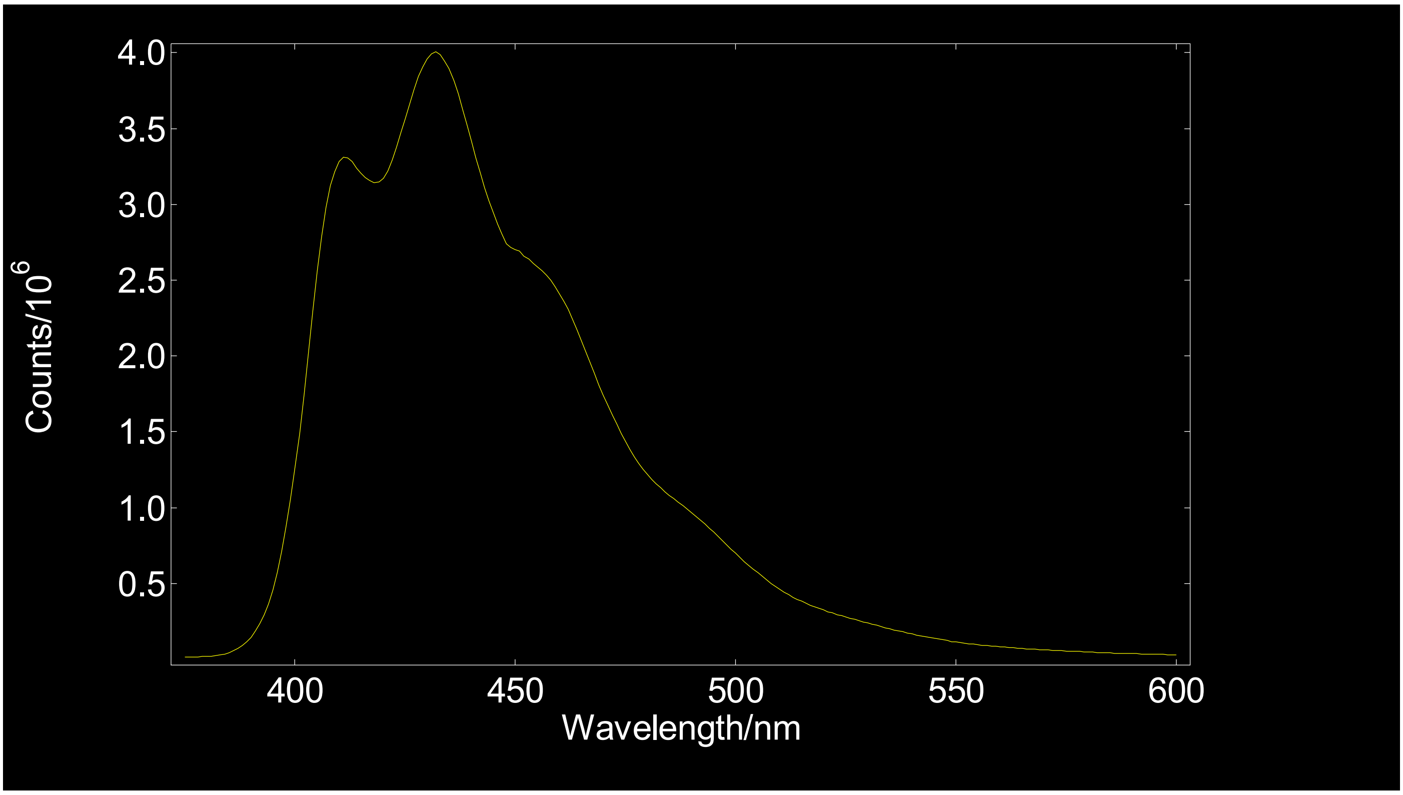

Figure S32. Emission spectrum of the compound **7b** in cyclohexane.

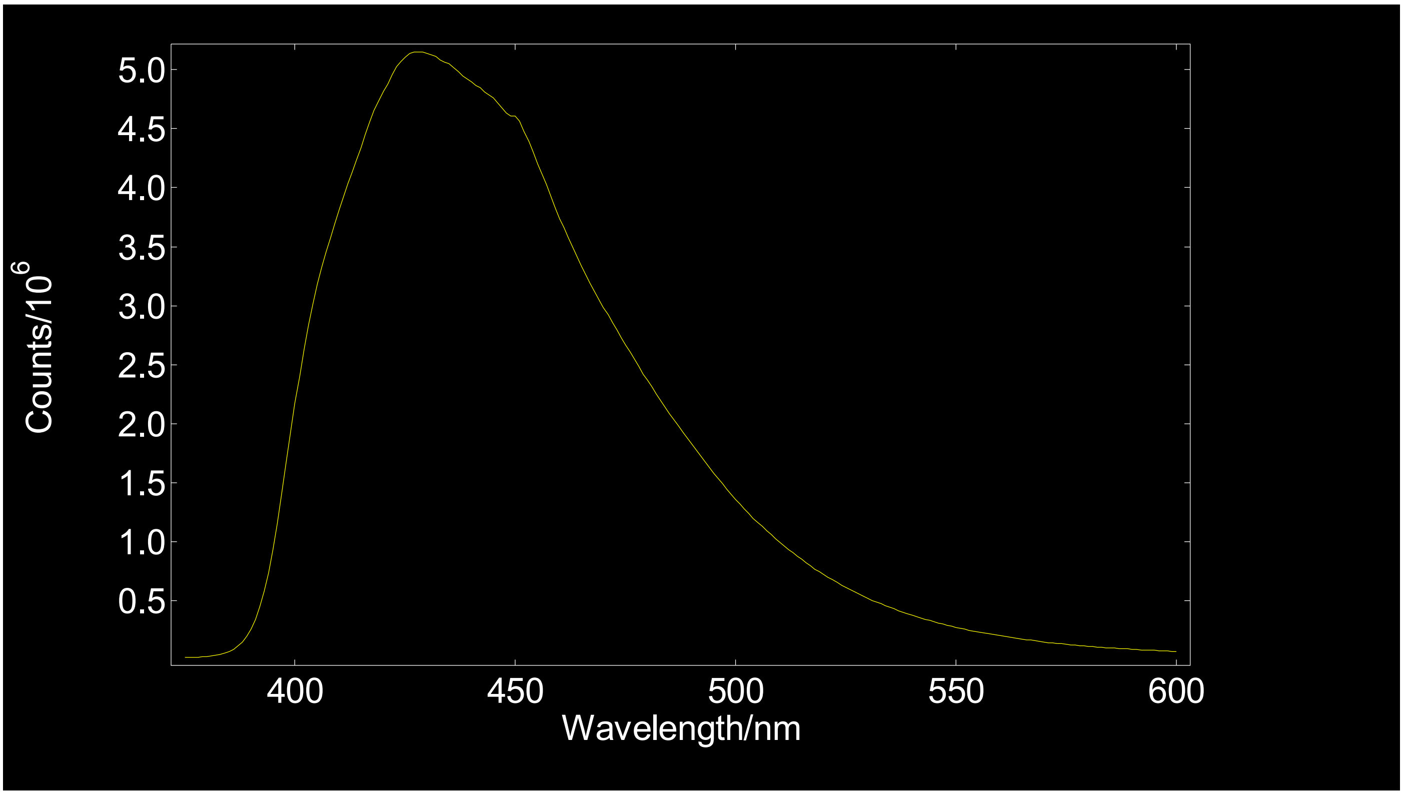

Figure S33. Emission spectrum of the compound **7c** in trichloromethane.

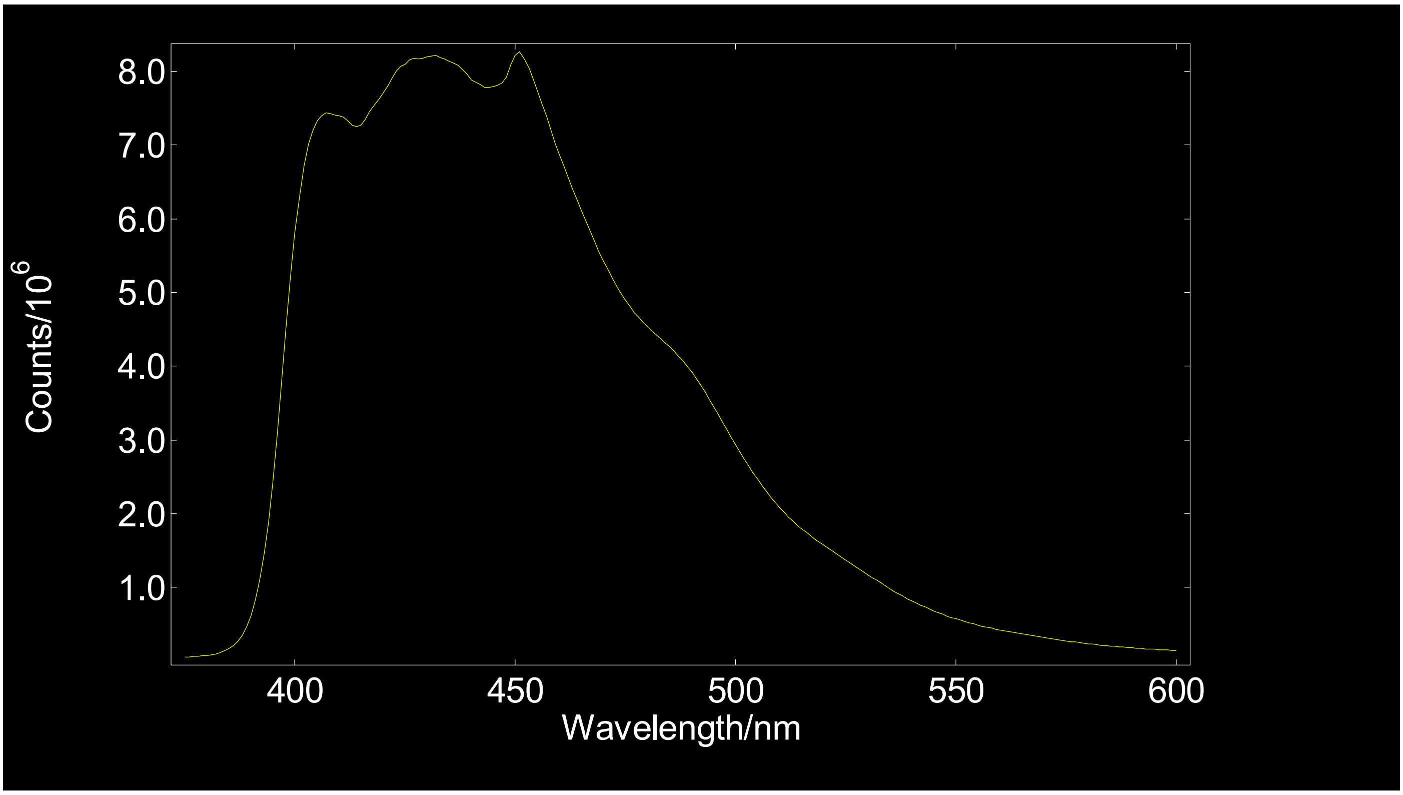

Figure S34. Emission spectrum of the compound **7c** in cyclohexane.

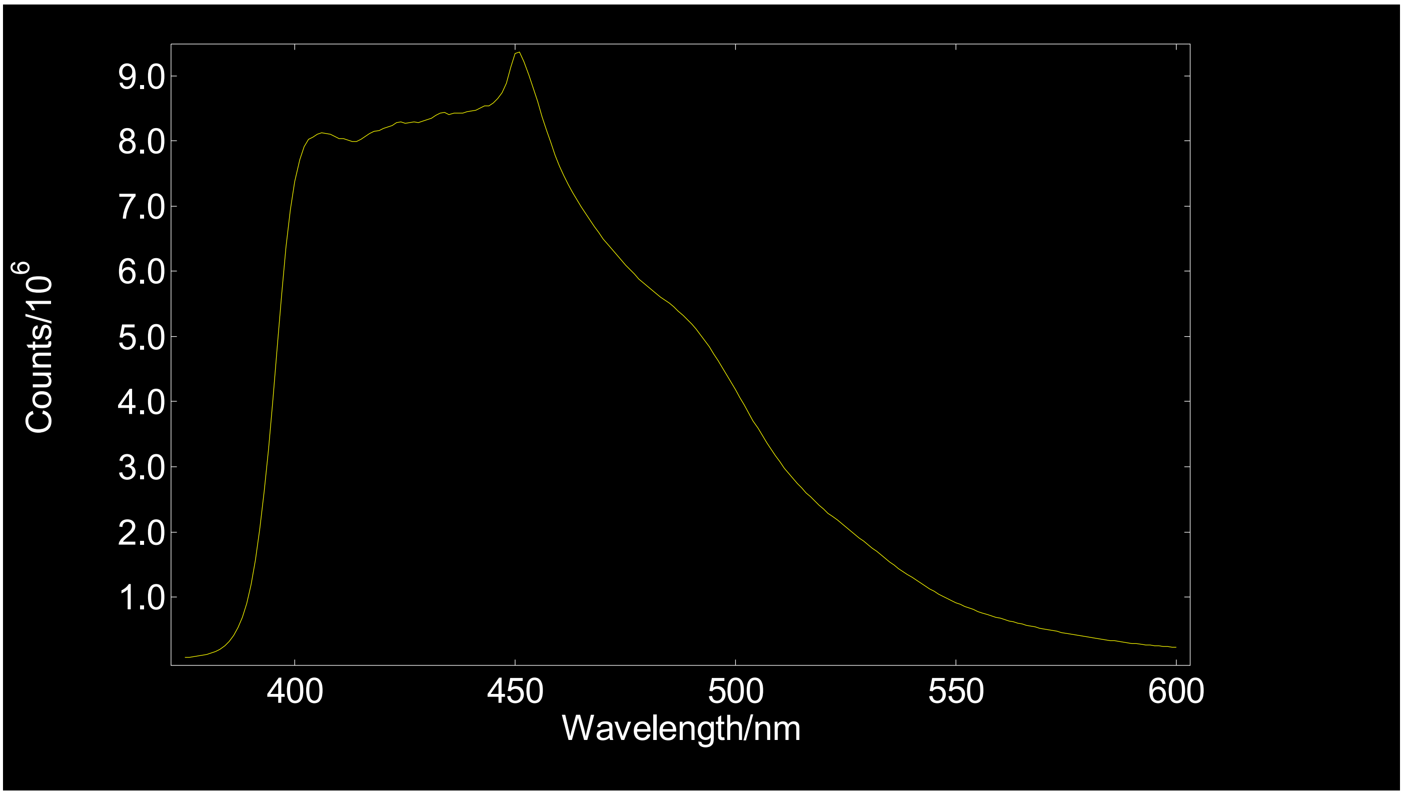

Figure S35. Emission spectrum of the compound **8c** in trichloromethane.

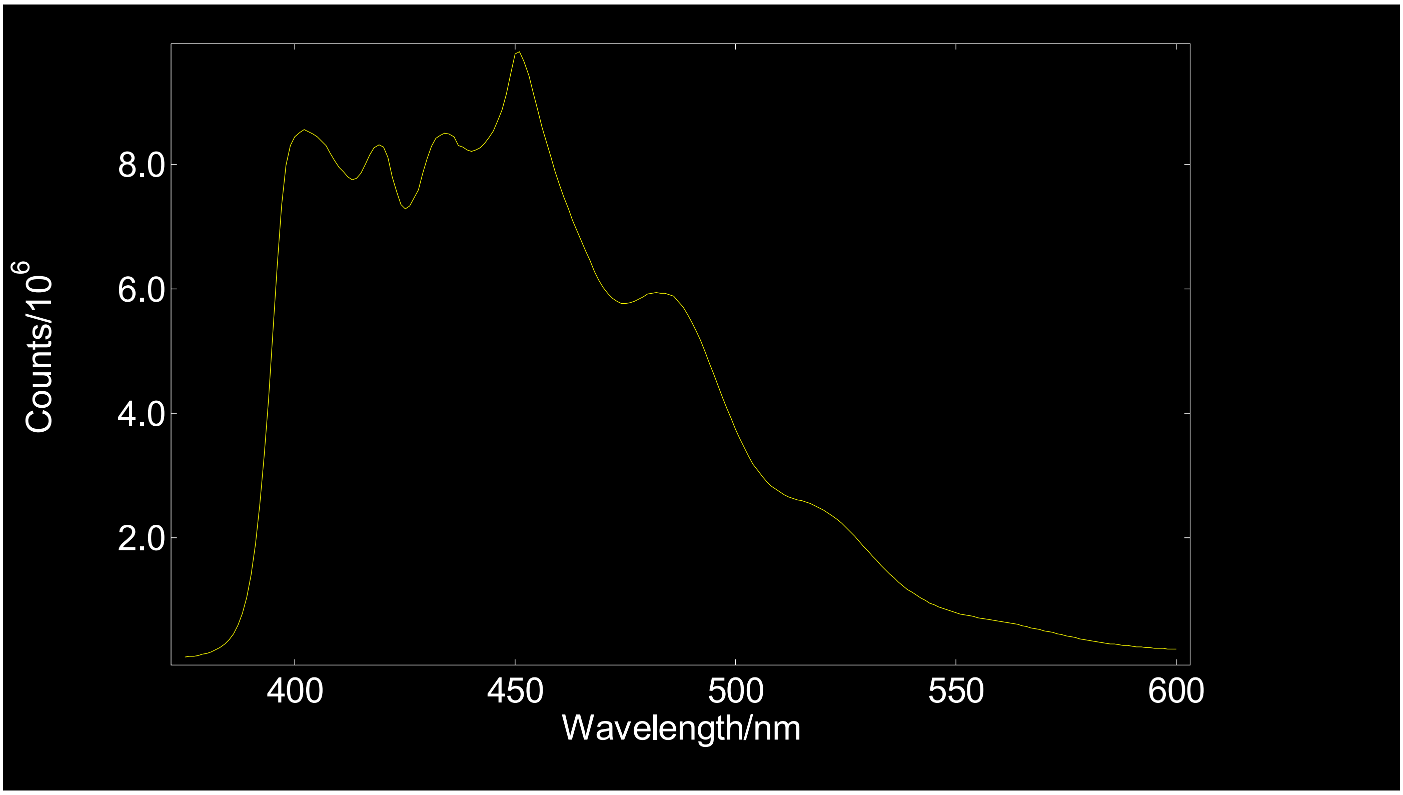

Figure S36. Emission spectrum of the compound **8c** in cyclohexane.

6. Absorption vs emission spectra of the obtained compounds

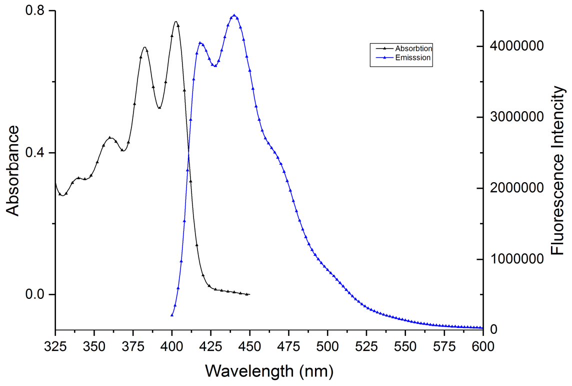

5a - trichloromethane

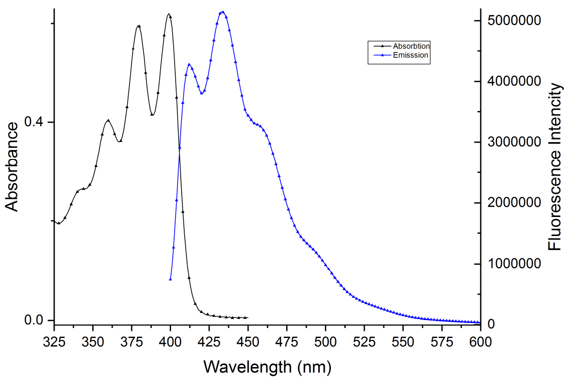

5a - cyclohexane

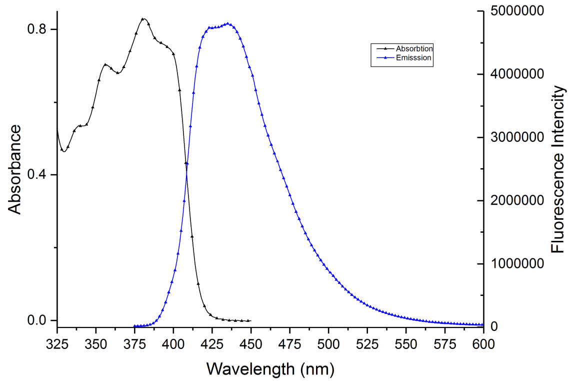

7a - trichloromethane

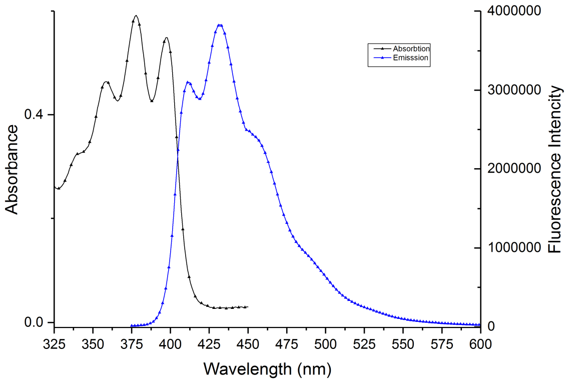

7a - cyclohexane

Figure S37. The absorption (black line) and emission (blue line) spectra of azasteroid derivatives **5a** and **7a** in trichloromethane (left column) and cyclohexane (right column). Excitation wavelength was: 396 nm for sample **5a** and 370 nm for samples **7b**.

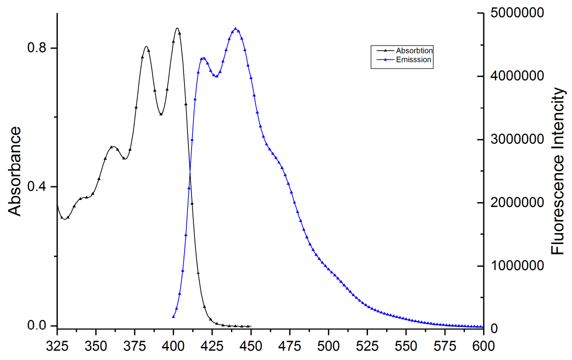

5b - trichloromethane

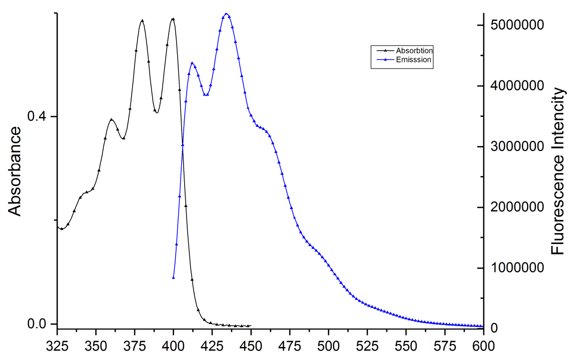

5b - cyclohexane

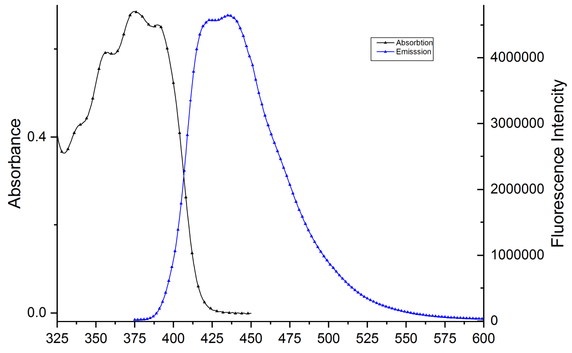

7b - trichloromethane

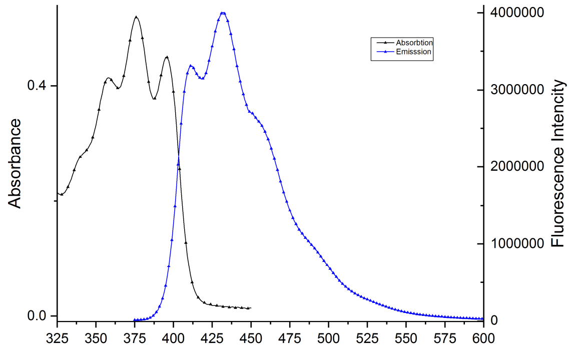

7b - cyclohexane

Figure S38. The absorption (black line) and emission (blue line) spectra of azasteroid derivatives **5b** and **7b** in trichloromethane (left column) and cyclohexane (right column). Excitation wavelength was: 396 nm for sample **5a** and 370 nm for samples **7b**.

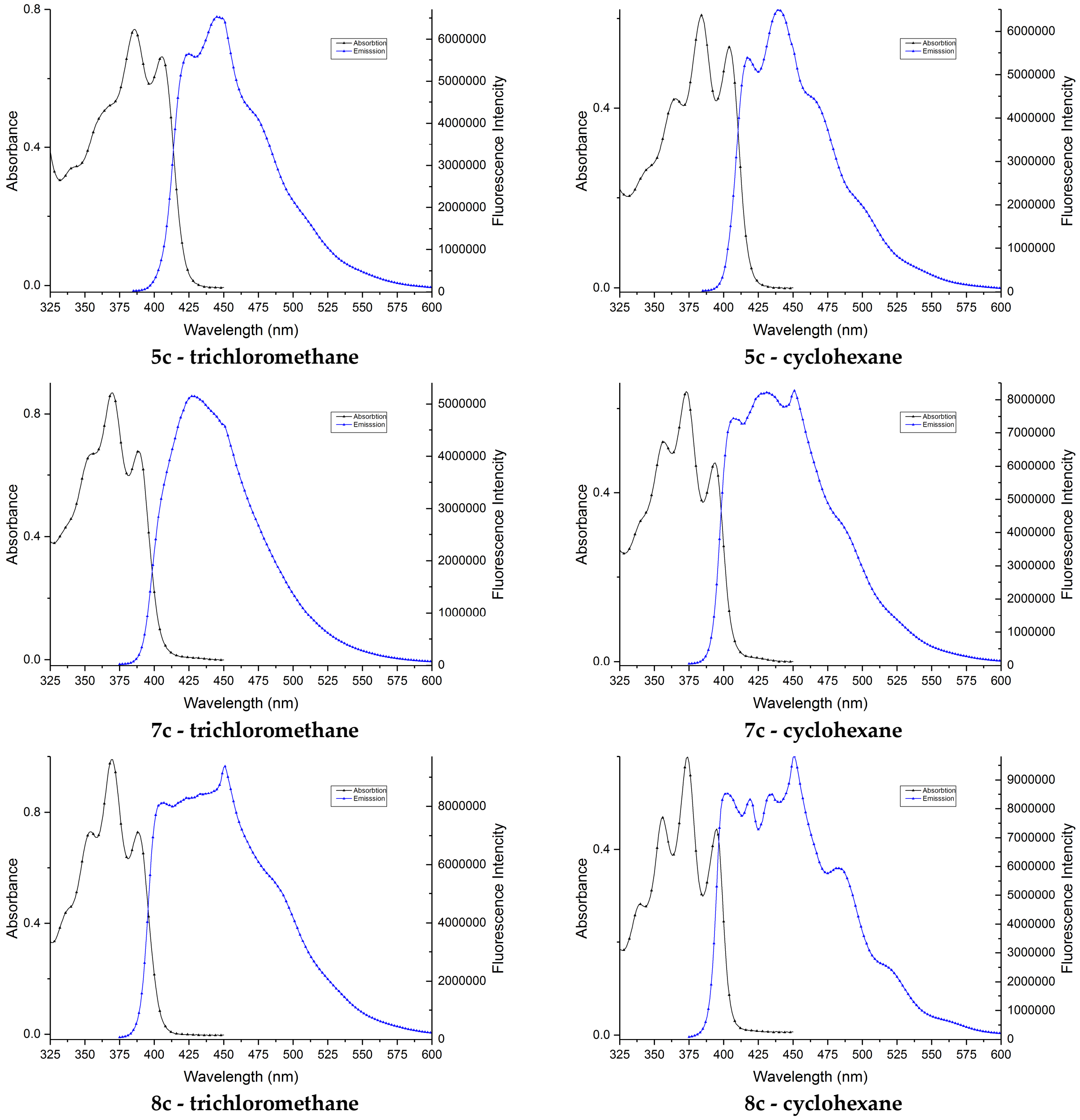

Figure S39. The absorption (black line) and emission (blue line) spectra of azasteroid derivatives **5c, 7c** and **8c** in trichloromethane (left column) and cyclohexane (right column). Excitation wavelength was: 380 nm for sample **5c** and 365 nm for samples **7c** and **8c**.
